# Anti-idiotypic antibodies elicit anti-HIV-1–specific B cell responses

**DOI:** 10.1084/jem.20190446

**Published:** 2019-07-25

**Authors:** Pia Dosenovic, Anna-Klara Pettersson, Abigail Wall, Eddy S. Thientosapol, Junli Feng, Connor Weidle, Komal Bhullar, Ervin E. Kara, Harald Hartweger, Joy A. Pai, Matthew D. Gray, K. Rachael Parks, Justin J. Taylor, Marie Pancera, Leonidas Stamatatos, Michel C. Nussenzweig, Andrew T. McGuire

**Affiliations:** 1 Laboratory of Molecular Immunology, The Rockefeller University, New York, NY; 2 Vaccine and Infectious Disease Division, Fred Hutchinson Cancer Research Center, Seattle, WA; 3 University of Washington University of Washington, Department of Global Health, Seattle, WA; 4 University of Washington University of Washington, Department of Immunology, Seattle, WA; 5 Howard Hughes Medical Institute, Chevy Chase, MD

## Abstract

Human anti-HIV-1 broadly neutralizing antibodies (bNAbs) protect against infection in animal models. However, bNAbs have not been elicited by vaccination in diverse wild-type animals or humans, in part because B cells expressing the precursors of these antibodies do not recognize most HIV-1 envelopes (Envs). Immunogens have been designed that activate these B cell precursors in vivo, but they also activate competing off-target responses. Here we report on a complementary approach to expand specific B cells using an anti-idiotypic antibody, iv8, that selects for naive human B cells expressing immunoglobulin light chains with 5–amino acid complementarity determining region 3s, a key feature of anti-CD4 binding site (CD4bs)–specific VRC01-class antibodies. In mice, iv8 induced target cells to expand and mature in the context of a polyclonal immune system and produced serologic responses targeting the CD4bs on Env. In summary, the results demonstrate that an anti-idiotypic antibody can specifically recognize and expand rare B cells that express VRC01-class antibodies against HIV-1.

## Abstract (precis)

We developed an anti-idiotypic antibody that specifically engages target B cells while mitigating off-target responses. This approach can be used as a vaccine strategy for variable pathogens where specific precursor B cells have been identified to correlate with protection.

## Graphical Abstract

**Figure:**
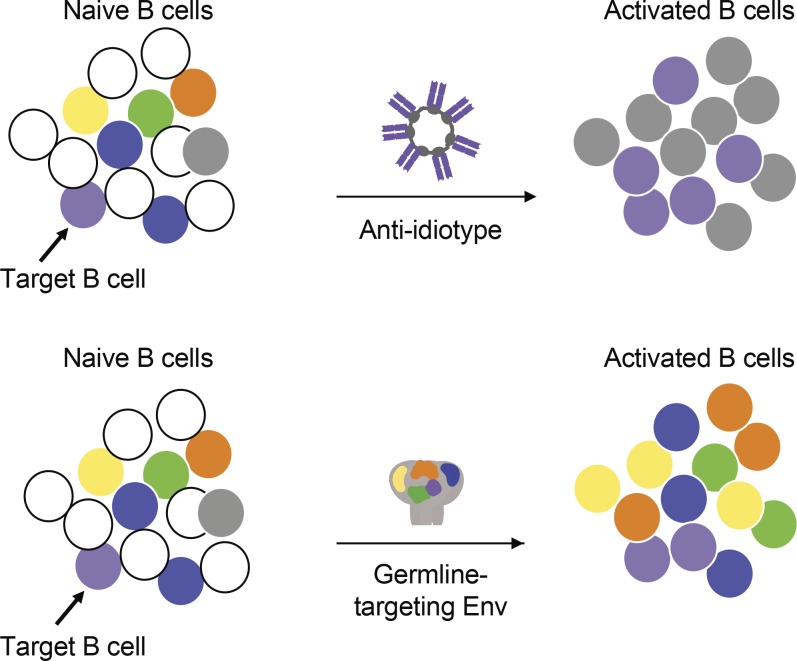

## Introduction

Despite nearly four decades of investigation, there is still no vaccine against HIV-1. However, broadly neutralizing antibodies (bNAbs) targeting conserved epitopes on the HIV-1 envelope (Env) are protective against infection by diverse viral strains in animal models (Burton and Hangartner, 2016; Haynes et al., 2016; Escolano et al., 2017; Kwong and Mascola, 2018). Thus, it is widely believed that a vaccine that elicits such antibodies would also be protective in humans.

VRC01-class antibodies are among the broadest and most potent bNAbs isolated to date. These antibodies share the same Ig heavy chain (IgH) V gene, VH1-2*02, which encodes a CDRH2 (complementary determining region 2, heavy chain) region that makes critical contacts with the CD4 binding site (CD4bs) on Env (Zhou et al., 2010, 2013, 2015; Diskin et al., 2011; Huang et al., 2016; Sajadi et al., 2018). In addition, these antibodies express Ig light chains (IgLs) with 5-aa-long CDRL3s (complementary determining region 3, light chain), a rare feature of human IgLs that is nonetheless required to accommodate the CDRH2-restricted mode of binding (West et al., 2012; Zhou et al., 2013).

An important impediment to vaccine development against HIV-1 is that immunization with soluble or multimerized recombinant Env proteins elicits antibodies that are type specific but show little or no neutralization breadth against heterologous viral variants (McCoy and Weiss, 2013; Sliepen and Sanders, 2016; Karlsson Hedestam et al., 2017; van Schooten and van Gils, 2018). This problem is not specific to HIV-1 but is also shared to a lesser extent with other variable pathogens such as influenza, Zika, and dengue virus (Brien et al., 2010; Fajardo et al., 2016; Wu and Wilson, 2017). Several different strategies have been devised to try to resolve this issue by focusing humoral responses on conserved epitopes. Examples include the development of scaffolds that carry the conserved epitopes (Ofek et al., 2010; Azoitei et al., 2011; Correia et al., 2014; Zhou et al., 2014), the use of short linear peptides that encompass target epitopes (Wang et al., 2010; Alam et al., 2017; Xu et al., 2018), shielding or deletion of off-target antigenic surfaces that are immunodominant (Barnett et al., 2001; Cherpelis et al., 2001; Mörner et al., 2009; Eggink et al., 2014; Yassine et al., 2015; Duan et al., 2018), and sequential immunization with different antigens that share the target epitope (Mörner et al., 2009; Guenaga et al., 2011).

An additional problem is that with few exceptions, the unmutated common ancestors of HIV-1 bNAbs fail to bind Env immunogens (Xiao et al., 2009; Huber et al., 2010; Zhou et al., 2010; Bonsignori et al., 2011, 2016; Ma et al., 2011; Scheid et al., 2011; Mouquet et al., 2012; Hoot et al., 2013; Jardine et al., 2013; Liao et al., 2013; McGuire et al., 2013, 2014b; Sok et al., 2013; Doria-Rose et al., 2014; Andrabi et al., 2015; Bhiman et al., 2015; Gorman et al., 2016; Stamatatos et al., 2017). To overcome this problem, Env proteins were specifically modified to engage bNAb precursors, an approach referred to as germline targeting (Jardine et al., 2013, 2016; McGuire et al., 2013; Steichen et al., 2016; Stamatatos et al., 2017). Successful germline targeting is dependent on competition between specific B cell precursors and off-target responses. Prime-boost regimens beginning with germline-targeting immunogens followed by sequential immunization with Envs intended to prime bNAb responses and shepherd the maturation to bNAbs have been evaluated for VRC01 class, the CH103 lineage, and PGT121-like bNAbs in human Ig knock-in mice (Briney et al., 2016; Escolano et al., 2016; Tian et al., 2016; Williams et al., 2017). Although this approach produced PGT121-like bNAbs in knock-in mice where bNAb precursor frequency is superphysiological, it has not yet been successful in diverse wild-type animals with polyclonal immune systems, possibly because of competition with off-target B cell responses (McGuire et al., 2014a, 2016; Dosenovic et al., 2015; Jardine et al., 2015; Briney et al., 2016; Sok et al., 2016; Tian et al., 2016; Medina-Ramírez et al., 2017; Vatti et al., 2017; Duan et al., 2018; Havenar-Daughton et al., 2018a,b).

To expand bNAb-producing B cells and their precursors and thereby give these cells a competitive advantage, we explored an alternative, non–Env-derived immunogen approach using a monoclonal anti-idiotypic antibody, iv8, that binds VRC01-class precursor B cell receptors (BCRs) with high affinity. When used as a prime, iv8 activated and expanded B cells expressing VRC01-class antibodies in a murine adoptive transfer system. Our results demonstrate that anti-idiotypic antibodies can expand VRC01-class B cells in vivo without activating B cell clones that produce off-target responses to Env.

## Results

### An anti-idiotypic antibody specific for VRC01-class bNAbs

**Figure 1.**
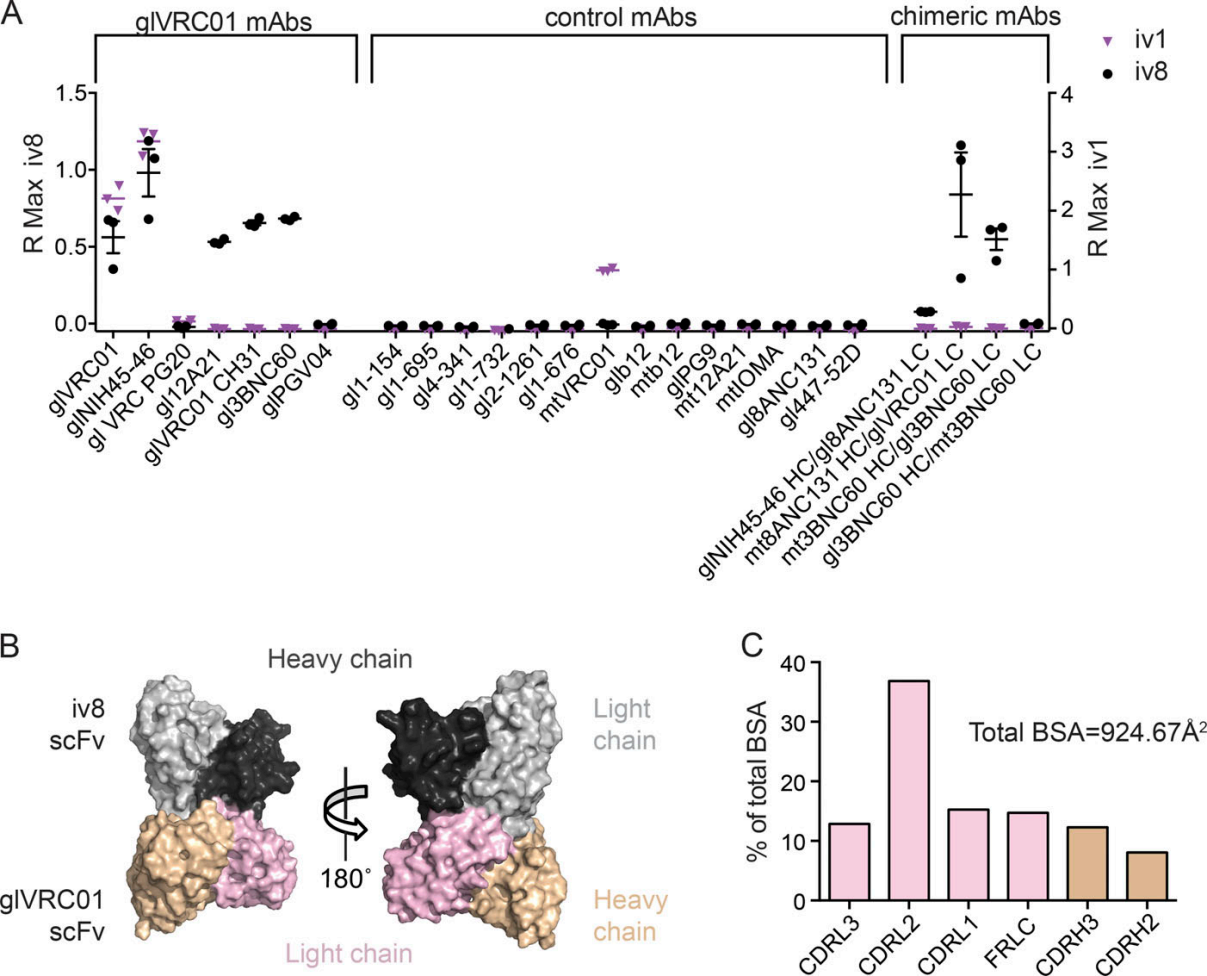
**iv8 binding to inferred germline VRC01-class BCRs. (A)** iv8 (black circles) and iv1 (purple triangles) binding to the indicated recombinant inferred-germline (gl), mutated (mt), or chimeric human monoclonal antibodies using BLI. Data points depict the maximum binding response (R Max) of the mAb following a 500-s association step. Each point represents the R Max of an independent replicate (*n* = 3). Lines indicate arithmetic means; error bars indicate SEM; HC and LC indicate IgHs and IgLs, respectively. **(B)** Structure of iv8 scFv bound to germline VRC01 scFv. The iv8 IgH and IgLs are shown in dark and light gray, respectively. Germline VRC01 IgH and IgLs are shown in beige and pink, respectively. **(C)** Distribution of BSA on glVRC01 upon iv8 binding. BSA on light-chain regions is shown in pink; BSA on heavy-chain regions is shown in beige.

We produced an anti-idiotypic monoclonal antibody against germline VRC01, iv8, using hybridoma technology (see Materials and methods). In addition to germline VRC01, iv8 binds other germline VRC01-class monoclonal antibodies, including NIH45-46, 3BNC60, 12A21, and VRC-CH31 (Scheid et al., 2011; Bonsignori et al., 2012). It does not bind to mature VRC01-class or all tested non–VRC01-class anti-HIV-1 bNAbs (Fig. 1 A). iv8 also bound a synthetic intermediate (SI) antibody comprising the mature 3BNC60 IgH and its germline IgL (3BNC60^SI^), but not to a 3BNC60 chimera composed of the germline IgH and mature IgL. Similarly, iv8 bound to a chimeric antibody comprising the germline 8ANC131 IgH (anti-CD4bs specific but derived from VH1-46) paired with germline VRC01 IgL, but not to germline NIH45-46 IgH paired with germline 8ANC131 IgL that contains a 9-aa CDRL3 (Fig. 1 A), indicating that binding mostly depends on VRC01-class germline light chains. Another idiotypic monoclonal antibody, iv1, bound to glVRC01 and glNIH45-46 and also bound weakly to mature VRC01. The more restricted binding of iv1 indicates that its binding depends at least partially on the CDRH3 (Fig. 1 A).

To determine the molecular basis for iv8 specificity, we solved the crystal structure of iv8 single-chain variable fragment (scFv) in complex with germline VRC01 scFv at 2.4 Å (Fig. 1 B and Table S1). Indeed, iv8 primarily interacts with the IgL of germline VRC01 (constituting 80% of the total buried surface area [BSA]) including CDRL3 residues (Tyr91, Glu96, and Phe97). Of the remaining germline VRC01 BSA; 60% of the contacts are with CDRH3 (Asp99, Tyr100, and Trp100B) and 40% with the germline-encoded VH1-2*02, including residues Trp47, Trp50, and Asn58 that make critical contacts with Env (Fig. 1, B and C; and Table S2; Zhou et al., 2010; West et al., 2012; Scharf et al., 2013). Together, the crystal structure and binding data establish the dominant role of the germline VRC01 light chain for recognition by the iv8 paratope.

### VRC01-class B cells respond to iv8 in a polyclonal immune system

**Figure 2.**
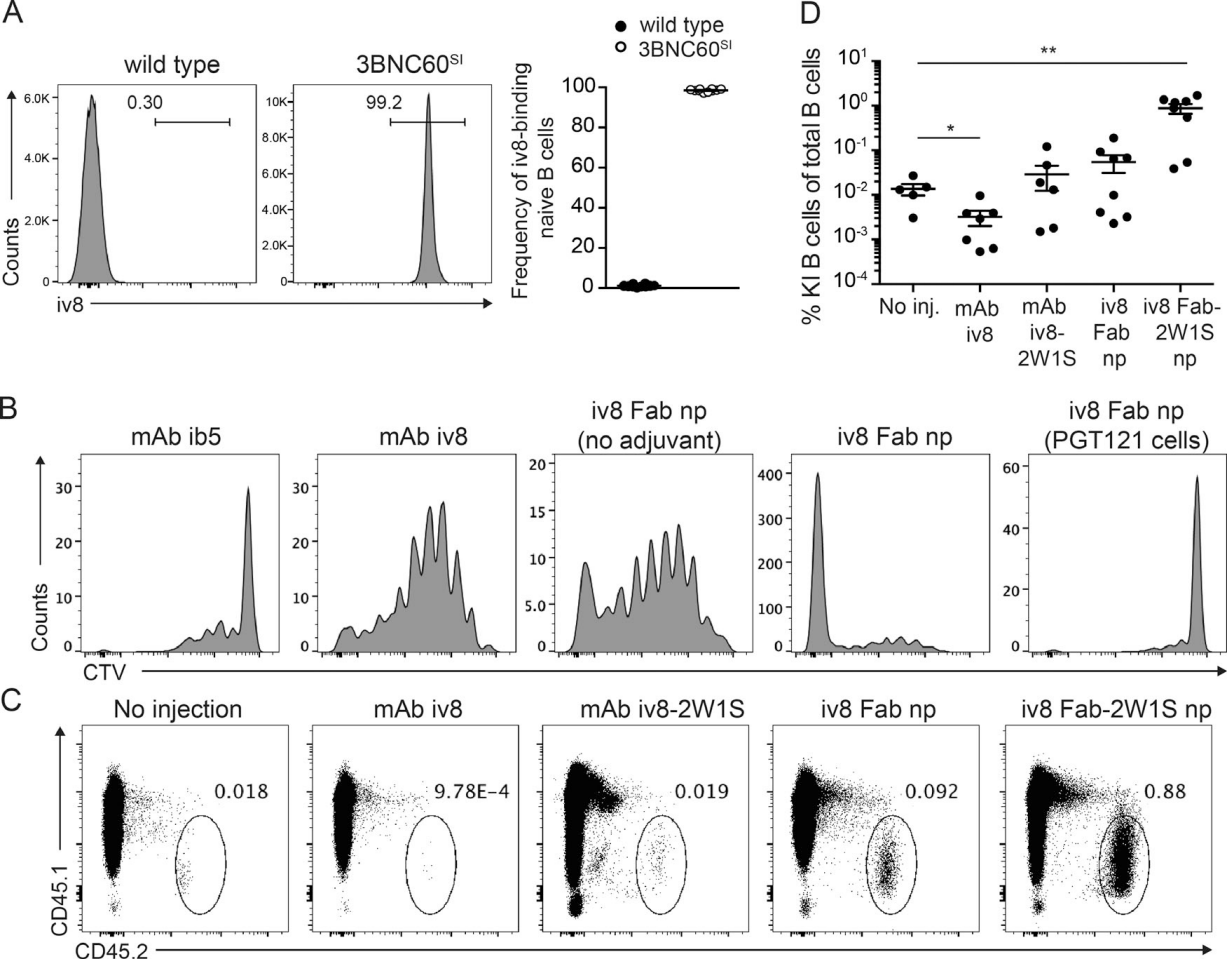
**Expansion of VRC01-class B cells in vivo. (A)** Representative flow cytometric plots of iv8-binding naive B cells from one wild-type mouse and one 3BNC60^SI^ mouse of 10 analyzed (left panels). Arithmetic mean frequency of iv8-binding naive B cells from 10 mice per group. Error bars indicate SEM (right). One representative experiment of three is shown. **(B)** Representative flow cytometry plots showing proliferation of adoptively transferred 3BNC60^SI^ cells as indicated by CTV dilution at 5 d after injection of (from left to right) control mAb (ib5, binds glb12 but not gl3BNC60) with Ribi, mAb iv8 with Ribi, iv8 Fab nanoparticles (np) without Ribi, iv8 Fab nanoparticles with Ribi, and CTV-labeled control PGT121 knock-in B cells in response to iv8 Fab nanoparticles with Ribi. One representative animal of four per group is shown from one experiment of three. **(C)** Flow cytometry plots pregated on total B cells (lymphocytes, singlets, dump^−^, live, B220^+^) indicating frequency of 3BNC60^SI^ knock-in cells of total B cells at 14 d after injection of the indicated constructs in Ribi adjuvant conjugated to 2W1S peptide or not. One representative animal of two to five per group in one experiment of three. **(D)** Frequency of 3BNC60^SI^ knock-in cells in mice injected as in C. Lines indicate arithmetic mean values of individual mice per group, with error bars indicating SEM of two replicate experiments with two to five mice per group per experiment. Statistics were calculated using the Mann–Whitney *U* test. *, P ≤ 0.05; **, P ≤ 0.01.

To determine whether iv8 binds to mouse B cells expressing VRC01-class antibodies, we isolated naive B cells from wild-type and 3BNC60^SI^ knock-in mice (Dosenovic et al., 2018). Whereas nearly all 3BNC60^SI^ knock-in B cells were positive for iv8 binding, the wild-type B cells were negative (Fig. 2 A).

To determine whether iv8 can activate VRC01-class B cells in the context of a polyclonal immune repertoire, we performed adoptive transfer experiments using 3BNC60^SI^ B cells (Dosenovic et al., 2018). To assess cell division of target B cells in vivo at early time points, an excess of naive 3BNC60^SI^ B cells (>2 × 10^6^) were labeled with CellTrace Violet (CTV) and transferred into wild-type congenic recipient mice. The mice were injected with a control anti-idiotypic antibody (ib5; Bancroft et al., 2019) in Ribi adjuvant, or with iv8 in Ribi adjuvant, or with a multimeric nanoparticle in which the iv8 fragment antigen binding (Fab) was fused to the N-terminus of a multimerization domain derived from the *Gallus gallus* complement component 4B (C4b) binding protein with or without Ribi adjuvant (Hofmeyer et al., 2013). 5 d after injection, there was no detectable cell division of 3BNC60^SI^ B cells in mice that received the negative control antibody ib5. In contrast, both intact iv8 (mAb iv8) and iv8 Fab nanoparticles induced strong proliferation of 3BNC60^SI^ B cells in vivo as measured by dilution of CTV fluorescence (Fig. 2 B). However, iv8 Fab nanoparticles were more efficient at inducing cell proliferation than mAb iv8, and the addition of Ribi adjuvant enhanced the effect (Fig. 2 B). Control PGT121 knock-in B cells, which recognize a glycopeptide epitope on the third variable region of Env, showed no measurable cell proliferation in response to iv8 Fab nanoparticles (Fig. 2 B; Escolano et al., 2016). We conclude that iv8 can induce proliferation of VRC01-class B cells in vivo in the context of a polyclonal repertoire, and that iv8 Fab nanoparticles are more efficient in doing so than the intact monoclonal antibody.

To investigate 3BNC60^SI^ B cell responses to iv8 at later time points, we injected iv8 into mice harboring ∼25,000 naive 3BNC60^SI^ B cells. We assayed the mice at 14 d after injection. Although the transferred B cells were readily detected at this time point in noninjected mice, they were significantly decreased and barely detectable in mice injected with intact iv8 (Fig. 2, C and D). To evaluate whether the observed loss of activated B cells was due to insufficient T cell help, recipient mice were preprimed with 2W1S peptide to elicit CD4^+^ helper T cells and injected with iv8 fused to the 2W1S peptide (mAb iv8-2W1S; Fig. 2, C and D) in Ribi (Rees et al., 1999; Moon et al., 2007). The frequency of 3BNC60^SI^ B cells in 2W1S peptide primed mice injected with mAb iv8-2W1S was comparable to that of unimmunized mice at day 14. In contrast, mice immunized with iv8 Fab nanoparticles with no 2W1S peptide showed no decrease in 3BNC60^SI^ B cells over controls. Conjugation of the 2W1S helper peptide to the C-terminus of the iv8 Fab nanoparticle increased the frequency of 3BNC60^SI^ B cells up to ∼65-fold over the controls, an improvement that we ascribe to the combination of multimerization and recruitment of preexisting T cell help (Fig. 2, C and D).

**Figure 3.**
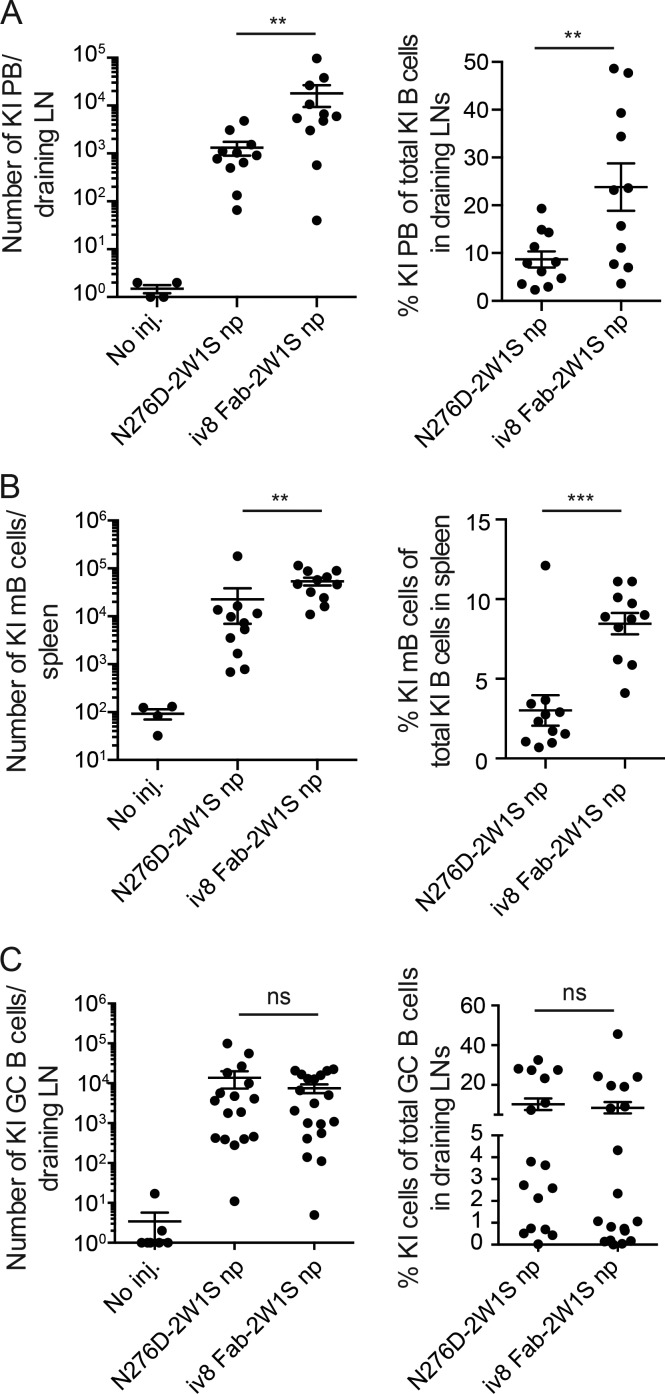
**B cell fates in response to iv8 Fab-2W1S or N276D-2W1S nanoparticles. (A)** Number of 3BNC60^SI^ knock-in (KI) plasmablasts (PB; CD138^+^B220^low^Env^high^; left) or frequency of 3BNC60^SI^ plasmablasts of total 3BNC60^SI^ cells (right) per draining LN in 2W1S-primed adoptively transferred wild-type mice 5 d after injection (inj.) with iv8 Fab-2W1S or N276D-2W1S nanoparticles (np) compared with noninjected controls. Lines indicate arithmetic means, and error bars indicate SEM. Each data point represents the average from two draining lymph nodes collected from a single animal (*n* = 4 uninjected, *n* = 11 N276D-2W1S np injected, *n* = 11 iv8 Fab-2W1S np injected). Merged data from two independent experiments are shown. **(B)** As in A, but measuring number of 3BNC60^SI^ early memory B cells (mB; B220^+^CTV^low^GL7^−^CD38^+^; left) or frequency of 3BNC60^SI^ early memory B cells of total 3BNC60^SI^ (right) in spleen at 5 d after injection. Lines indicate arithmetic means, with error bars indicating SEM. Each data point represents the analysis in spleen of one animal (*n* = 4 uninjected, *n* = 11 N276D-2W1S np injected, *n* = 11 iv8 Fab-2W1S np injected). Merged data from two independent experiments. **(C)** As in A, but measuring 3BNC60^SI^ GC B cells (B220^+^CD95^+^CD38^−^; left) and frequency of 3BNC60^SI^ cells of total GCs (right) 14 d after injection. Lines indicate arithmetic means, and error bars indicate SEM. Merged data from three independent experiments are shown. Each data point represents the average number from two draining lymph nodes collected from a single animal (*n* = 7 uninjected, *n* = 17 N276D-2W1S np injected, *n* = 19 iv8 Fab-2W1S np injected). Statistics in A–C were calculated using the Mann–Whitney *U* test. **, P ≤ 0.01; ***, P ≤ 0.001; ns, nonsignificant.

Next, we compared the fate of 3BNC60^SI^ B cells in response to injection with iv8 or an authentic HIV-1 Env-derived germline-targeting immunogen, 426c-N276D, which has an affinity of ∼40 µM compared with ∼2 nM for iv8 for 3BNC60^SI^ (Table S3; McGuire et al., 2013; Dosenovic et al., 2018). 3BNC60^SI^ B cells were labeled with CTV and transferred into 2W1S-primed wild-type mice. Recipients were injected with 426c-N276D-2W1S nanoparticles or iv8 Fab-2W1S nanoparticles and assayed for plasmablast and early B cell memory formation 5 d later by flow cytometry. We observed an increased population of 3BNC60^SI^ B cells after iv8 Fab-2W1S nanoparticle injection, with an arithmetic mean of 1.8 × 10^4^ plasmablasts and 5.4 × 10^4^ early memory B cells compared with 1.0 × 10^3^ plasmablasts and 2.2 × 10^4^ early memory B cells in mice injected with 426c-N276D-2W1S nanoparticles. In contrast, these cells were barely detectable in unimmunized control mice (Fig. 3, A and B, left panels; and Fig. S1, A and B). Similar results were obtained when analyzing the relative frequency of 3BNC60^SI^-derived plasmablasts and early memory B cells (Fig. 3, A and B, right panels).

Germinal center (GC) development was also assayed 14 d after injection using the same experimental setup. Draining lymph nodes contained an arithmetic mean of 1.4 × 10^4^ and 8 × 10^3^ 3BNC60^SI^-derived GC B cells after 426c-N276D-2W1S or iv8 Fab-2W1S nanoparticle injection, respectively (Fig. 3 C, left panel; and Fig. S1 C). The 3BNC60^SI^ GC cells constituted 11% and 8% of all GC cells after 426c-N276D-2W1S or iv8 Fab-2W1S nanoparticle injection, respectively (Fig. 3 C, right panel, and Fig. S1 C). In comparison, 3BNC60^SI^ GC cells were difficult to detect in unimmunized controls (Fig. 3 C, left). We conclude that injection with 426c-N276D-2W1S or iv8 Fab-2W1S nanoparticles induces similar levels of GC responses by 3BNC60^SI^ B cells. However, iv8 Fab-2W1S nanoparticles induce more plasmablast and early memory B cell responses than 426c-N276D-2W1S nanoparticles.

### Antibody responses in serum after injection with iv8 and Env

To evaluate serum antibody responses, we injected 2W1S-primed wild-type recipient mice harboring ∼25,000 naive 3BNC60^SI^ B cells with 426c-N276D-2W1S or iv8 Fab-2W1S nanoparticles. Serum antibody responses were evaluated by ELISA using eOD-GT8, which is a distinct HIV-1 Env–derived immunogen (Jardine et al., 2013, 2015; Dosenovic et al., 2015).

**Figure 4.**
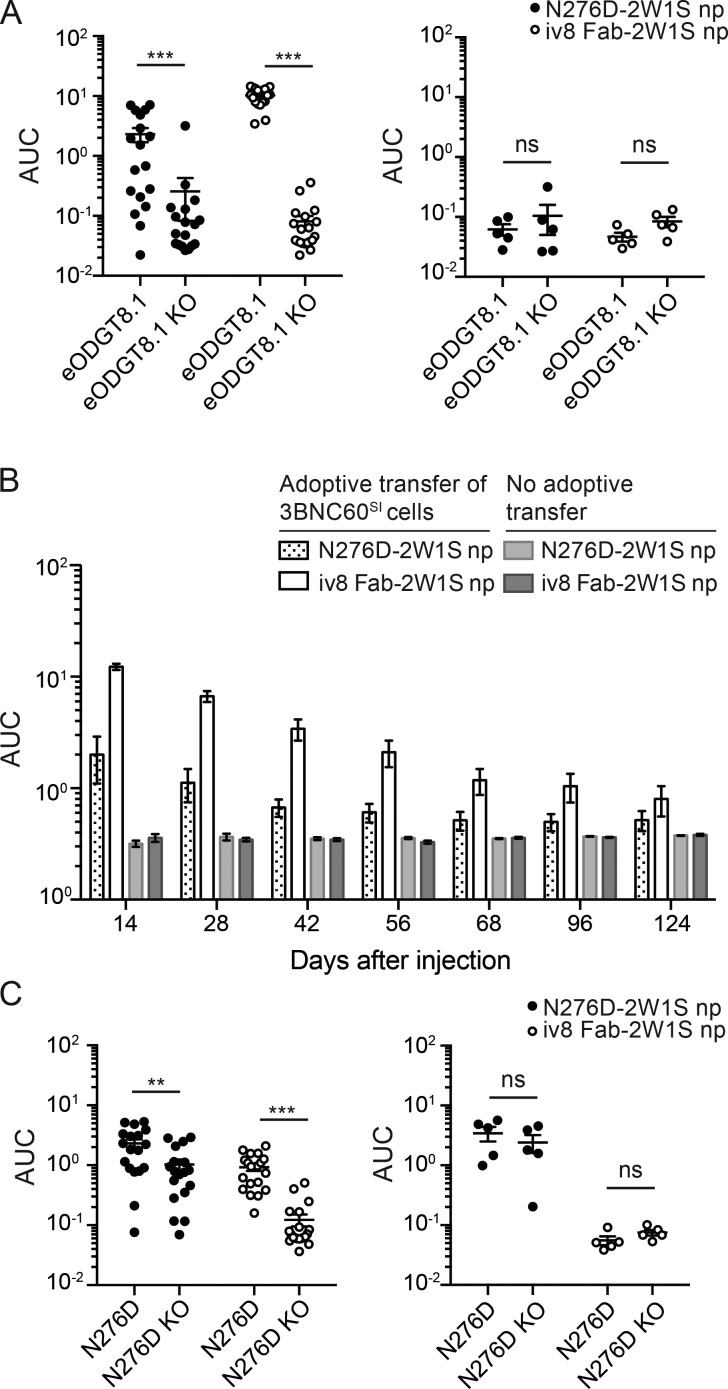
**Serum antibody titers. (A)** Serum antibody titers against eOD-GT8 and eOD-GT8-KO in 2W1S-primed wild-type mice at 14 d after injection with Env N276D-2W1S or iv8 Fab-2W1S nanoparticles (np) in the presence (left panel) or absence (right panel) of adoptively transferred 3BNC60^SI^ cells. Merged data from three independent experiments is shown with five to nine mice per group (left panel; *n* = 18 N276D-2W1S np injected, *n* = 20 iv8 Fab-2W1S np injected, right panel; *n* = 5 N276D-2W1S np injected, *n* = 5 iv8 Fab-2W1S np injected). Lines indicate arithmetic means. Error bars indicate SEM. **(B)** Serum antibody titers against eOD-GT8 from mice injected as in A at time points between 14 and 124 d after injection. Merged data from two independent experiments. Bars indicate arithmetic mean, and error bars indicate SEM. Merged data from two independent experiments are shown with two to five mice per group in each experiment (*n* = 10 N276D-2W1S np injected, *n* = 10 iv8 Fab-2W1S np injected). **(C)** ELISA using serum from same animals in A, to measure responses to Env N276D (N276D) and Env N276D KO (N276D KO). Statistics in A and C were calculated using the Mann–Whitney *U* test. **, P ≤ 0.01; ***, P ≤ 0.001; ns, nonsignificant. AUC, area under the curve.

Mice receiving 3BNC60^SI^ B cell transfers developed eOD-GT8–binding serum antibodies in response to both 426c-N276D-2W1S and iv8 Fab-2W1S nanoparticles, but control mice did not (Fig. 4 A). The eOD-GT8 responses were specific for the VRC01 epitope (the CD4bs) because there was minimal reactivity to an epitope specific knockout (eOD-GT8-KO; Fig. 4 A).

Adoptively transferred mice injected with iv8 Fab-2W1S or 426c-N276D-2W1S nanoparticles showed eOD-GT8–specific antibody responses for 2–3 mo (Fig. 4 B). However, 426c-N276D-2W1S nanoparticles also elicited significant off-target responses to HIV-1 Env that were not observed after iv8 Fab-2W1S nanoparticle injection (Fig. 4 C). We conclude that iv8 injection engages target B cells and elicits CD4bs-specific antibody responses with minimal off-target responses to Env.

### iv8 selects B cells with VRC01-class IgLs

To determine whether iv8 can select VRC01-class B cells in vivo, we used knock-in mice expressing only the germline or mature IgHs of the human 3BNC60 antibody (3BNC60 HCgl [heavy-chain germline] or 3BNC60 HCmt [heavy-chain mutated], respectively). Naive B cells in these mice express the 3BNC60 knock-in IgH paired with mouse IgLs (Dosenovic et al., 2015). To ensure that stimulated B cells would receive adequate T cell help, the mice were preprimed with the 2W1S peptide.

**Figure 5.**
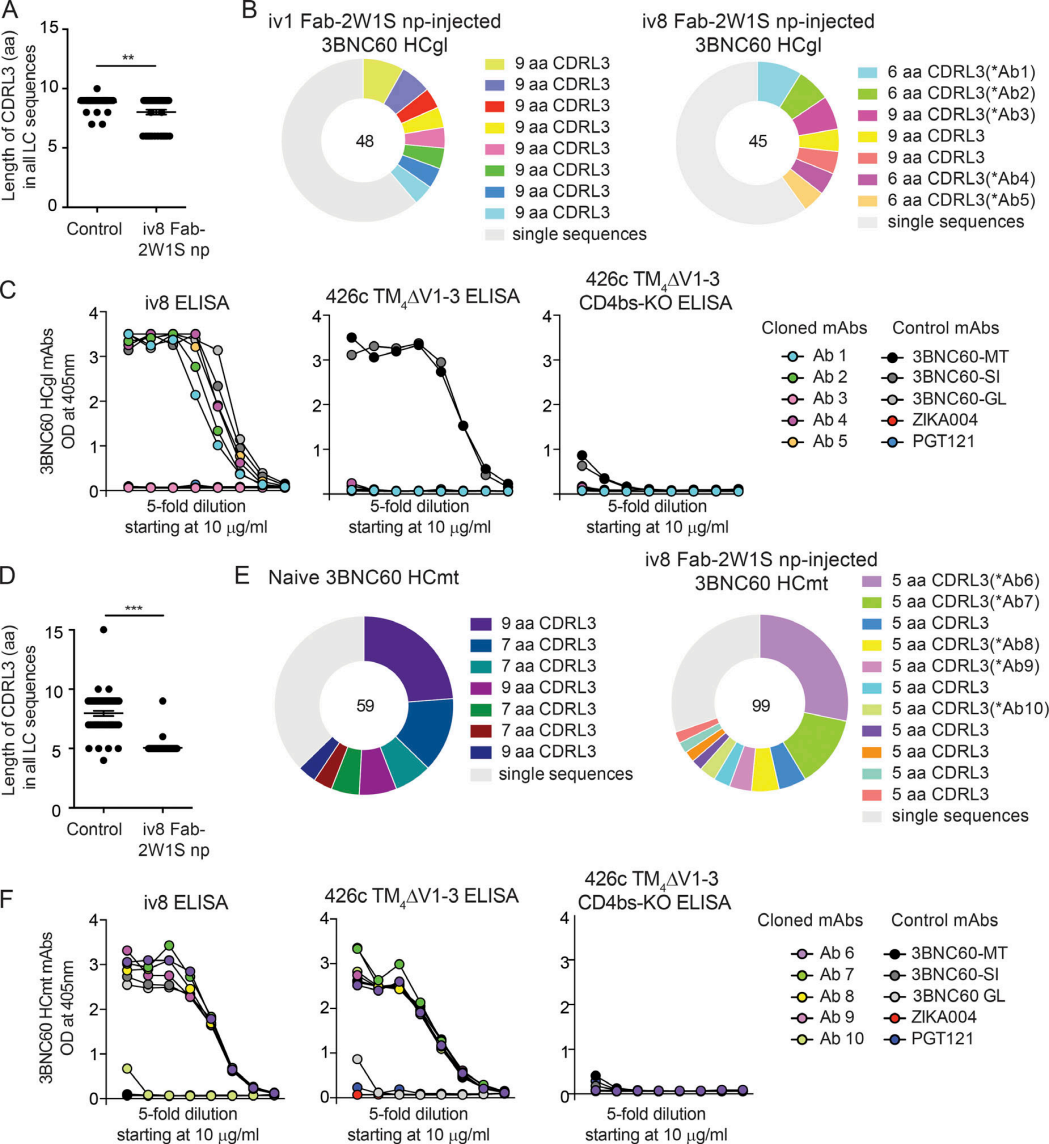
**Selection for VRC01-class IgLs by iv8 in heavy-chain knock-in mice. (A)** CDRL3 lengths in amino acids of BCRs from single-cell sorted IgG1^+^ GC B cells (B220^+^CD95^+^CD38^−^IgG1^+^) from two control-injected (iv1 Fab-2W1S nanoparticles [np], which bind glVRC01 and glNIH45-46 but not gl3BNC60) and two iv8 Fab-2W1S np-injected 2W1S-primed 3BNC60 HCgl mice obtained 14 d after injection. Lines indicate arithmetic means; error bars indicate SEM. **(B)** Pie charts indicate B cell clones from sequences obtained in A. Each color represents a clone. Light gray indicates single sequences obtained from nonclonal B cells. Numbers in the middle of the pie chart indicate the total number of sequences analyzed. **(C)** Representative monoclonal antibodies corresponding to the BCR sequences determined in B (Ab1–Ab5) were tested in ELISA against iv8 (left), 426cTM_4_ΔV1-3 (middle), or 426cTM_4_ΔV1-3 CD4bs-KO (right). Graphs are representative of two independent experiments. **(D)** Graph indicating CDRL3 lengths in amino acids of BCRs from single-cell sorted naive and memory B cells purified on the basis of 426c TM4ΔV1–3 antigen binding. Memory cells (B220^+^IgM^−^CD38^+^IgG1^+^) were obtained from 2W1S-primed 3BNC60 HCmt mice injected with iv8 Fab-2W1S nanoparticles 42 d after injection. Lines indicate arithmetic means, and error bars indicate SEM. **(E)** Pie charts (visualized as in B) indicate sequenced B cell clones obtained in D. **(F)** Representative monoclonal antibodies corresponding to the BCR sequences determined in E (Ab6–Ab10), were tested by ELISA for binding to iv8 (left), 426cTM_4_ΔV1-3 (middle), or 426cTM_4_ΔV1-3 CD4bs-KO (right). Graphs are representative of two independent experiments. 3BNC60-MT, 3BNC60-SI, and 3BNC60-GL mAbs are included as controls, and ZIKA004 and PGT121 are included as negative mAb controls in C and F. Statistics were calculated using the Mann–Whitney *U* test in A and D. **, P ≤ 0.01; ***, P ≤ 0.001. LC, light chain.

Single IgG1^+^ GC B cells were purified from draining lymph nodes of iv8 Fab-2W1S nanoparticle-injected and control-injected (iv1 Fab-2W1S nanoparticles) 3BNC60 HCgl mice, and their IgLs were sequenced (Fig. S2 A). These cells were not selected based on Env binding, because the 3BNC60 HCgl antibodies do not bind to Env with high affinity. When all IgLs were considered, there was a significant preference for shorter CDRL3s in iv8 Fab-2W1S nanoparticle–injected mice (P = 0.0014; Fig. 5 A and Tables S4 and S5). This result was also seen in expanded B cell clones (Fig. 5 B and Tables S4 and S5). Four of five recombinant, soluble IgGs corresponding to BCRs from five representative expanded 3BNC60 HCgl clones with 6-aa CDRL3s (Table S7) bound strongly to iv8 (Fig. 5 C). None of these mAbs bound to the 426cTM_4_ΔV1-3 or eOD-GT8 germline-targeting immunogens (Fig. 5 C).

IgL sequences were also obtained from single Env-binding 3BNC60 HCmt B cells in naive or iv8 Fab-2W1S nanoparticle-injected mice. When comparing all sequences, we found that iv8 selected for expansion of 3BNC60 HCmt memory B cells with significantly shorter CDRL3s compared with B cells of naive mice (P < 0.0001; Fig. 5 D and Tables S6 and S7). Moreover, all of the IgG1^+^ memory B cell clones obtained from two 3BNC60 HCmt mice injected with iv8 Fab-2W1S nanoparticles expressed IgLs with 5-aa-long CDRL3 sequences (Fig. 5 E and Tables S6 and S7). Four of five recombinant, soluble IgGs corresponding to BCRs from five representative expanded 3BNC60 HCmt clones (Table S7) bound strongly to iv8, and all bound to the 426cTM_4_ΔV1-3 germline-targeting immunogen in a CD4bs-specific manner (Fig. 4 F). None of the cloned mAbs bound to eOD-GT8 (not depicted). We conclude that iv8 selects for B cells with shorter-than-average CDRL3s in 3BNC60 HC knock-in mice, and that iv8-responding B cells from 3BNC60 HCmt mice with VRC01, like 5-aa CDRL3, cross-react with a germline-targeting Env.

### iv8 recognizes B cells expressing antibody light chains similar in length to VRC01-class antibodies in human peripheral blood

**Figure 6.**
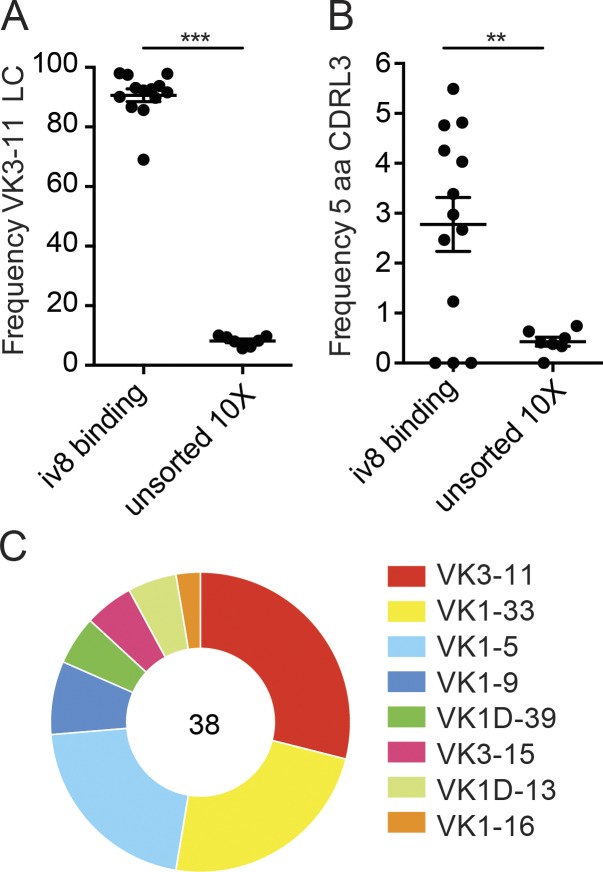
**iv8-binding to human peripheral blood cells.** Single naive human B cells were purified by cell sorting with fluorescently labeled iv8, and the IgL kappa transcripts were recovered by RT-PCR and sequenced or subjected to paired BCR sequencing using the chromium platform from 10X Genomics. As a control, naive human B cells were enriched using magnetic beads, and paired BCR sequencing was performed using the chromium platform. **(A)** Frequency of IgL kappa transcripts derived from VK3-11 in iv8^+^ or total B cells. Each dot represents one iv8^+^ sort (*n* = 13) or paired chromium sequencing run of magnetic bead–enriched (total naive) B cells (*n* = 7). **(B)** Frequency sequenced IgL kappa transcripts with a 5-aa CDLR3 in iv8^+^ or total B cells. Each dot represents one iv8^+^ sort (*n* = 13) or chromium sequencing run of magnetic bead–enriched (total naive) B cells (*n* = 7). Lines in A and B indicate arithmetic means, and error bars indicate SEM. **(C)** IgVK gene usage for of iv8^+^ B cells with 5-aa CDRL3s identified in B. Number in the middle of the pie chart indicates the total number of IgL kappa sequences analyzed. Statistics were calculated using two-tailed unpaired Student’s *t* tests in A and B. **, P ≤ 0.01; ***, P ≤ 0.001. LC, light chain.

To determine if iv8 binds to naive human B cells expressing VRC01-class antibodies, we purified iv8^+^ peripheral blood B cells from seven HIV-1 negative donors and analyzed their IgHs and/or IgLs by RT-PCR (Tiller et al., 2008) or high-throughput paired sequencing on the chromium platform from 10X Genomics. More than 80% of the iv8^+^ B cells expressed the VK3-11 light chain found in VRC01 (Fig. 6 A). This is consistent with the extensive interactions found between iv8 and the glVRC01 light chain in the crystal structure (Fig. 1, B and C). In contrast, the overall frequency of VK3-11 in naive B cells was ∼8% (P < 0.0001; Fig. 6 A). In addition, an arithmetic mean of 2.8% of the iv8^+^ B cells expressed a light chain containing a 5-aa CDRL3, corresponding to a significant enrichment over the unsorted naive cells (P = 0.0056; Fig. 6 B). Although iv8 strongly selected B cells that express a VK3-11 light chain, only an arithmetic average of 29% of the light chains that expressed a 5-aa CDRL3 were derived from VK3-11 (Fig. 6 C). As might be expected from the structural information, the IgHs were not enriched for VH1-2. Thus, iv8 enriches for human B cells expressing antibodies with a VK3-11 light chains and 5-aa CDRL3s.

## Discussion

The relatively poor reactivity of inferred germline precursors of VRC01-class antibodies with conventional recombinant HIV-1 Env proteins led to the development of novel Env-derived immunogens that engage B cells expressing these antibodies (Scheid et al., 2011; Jardine et al., 2013, 2016; Klein et al., 2013; McGuire et al., 2013, 2016; Medina-Ramírez et al., 2017). However, germline targeting alone is not sufficient to elicit mature VRC01-class antibodies.

To overcome the glycan barrier to the CD4bs at position N276, the precursors of VRC01-class antibodies undergo prolonged affinity maturation and extensive somatic hypermutation in GCs (Liao et al., 2013; Doria-Rose et al., 2014). Under physiological conditions, this process involves an arms race between the virus and the humoral immune system in which antibody-mediated selection of viral mutants over a period of 1–3 yr leads to emergence of one or more bNAbs (Mascola and Haynes, 2013; Burton and Hangartner, 2016; Escolano et al., 2017; Landais and Moore, 2018). This process has been reproduced in knock-in mice expressing the inferred germline precursors of bNAbs targeting the base of the third variable region of Env and, in part, for VRC01-class antibodies, by sequential immunization (Dosenovic et al., 2015; Briney et al., 2016; Escolano et al., 2016; Tian et al., 2016).

However, the B cell compartment in knock-in mice is dominated by cells expressing inferred germline bNAbs, and there is little if any competition with other B cells. Effective competition between bNAb precursors and off-target B cells depends on their relative affinity to the immunogen and their frequency in the population (Dal Porto et al., 2002; Shih et al., 2002; Abbott et al., 2018; Dosenovic et al., 2018). The inferred germline precursor B cells of VRC01-class antibodies are rare in the normal human repertoire, and they would have to compete with a diverse set of more common B cells that recognize potentially irrelevant parts of the immunogen. Consistent with this idea, the 426c-derived germline-targeting immunogen elicited significant off-target anti-Env responses that compete with and may ultimately interfere with bNAb development (Dosenovic et al., 2015; Briney et al., 2016; McGuire et al., 2016; Tian et al., 2016; Duan et al., 2018).

We have tested the notion that anti-idiotypic antibodies can selectively expand rare B cells to increase the probability that such cells would contribute to the immune response. Several modifications were required to enhance the activity of iv8 in vivo. Whereas injection of the intact antibody led to targeted B cell depletion, a multimerized Fab fragment was a potent inducer of cell division, GC, early memory, and plasma cell development. These responses were further improved by preexisting T cell help, which was provided by priming with, and then adding, a T cell epitope to iv8.

**Figure 7.**
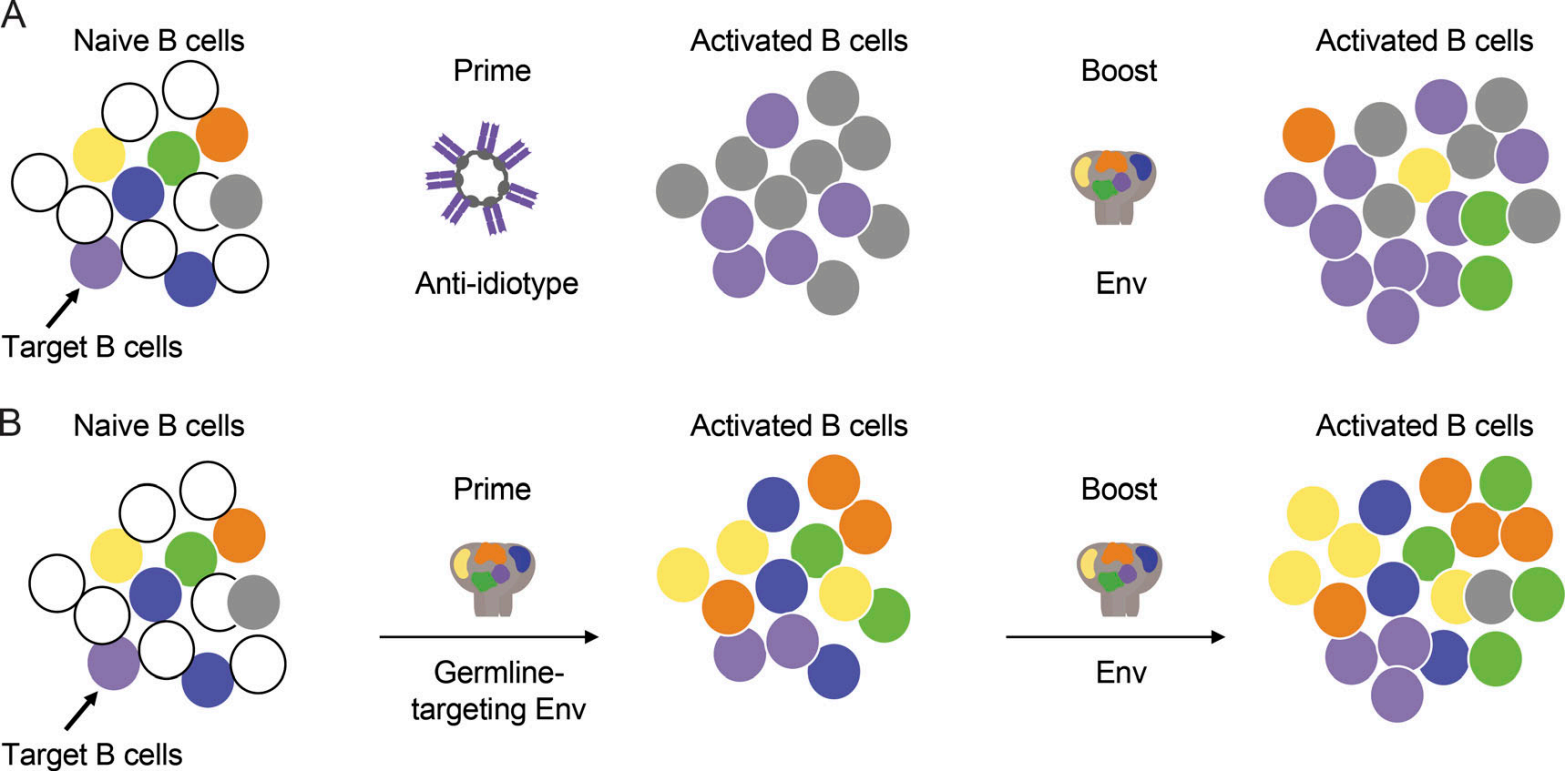
**Model for an anti-idiotype-prime/Env-boost regimen. (A)** An initial pool of B cells including off-target Env-specific B cells (yellow, green, orange, and blue), on-target Env-specific bNAb precursor B cells (purple), off-target anti-idiotypic antibody-specific B cells directed against the multimerization domain and constant regions (gray), and irrelevant B cells (white; left). Activated B cell responses to immunization with an anti-idiotype prime that expands bNAb precursor B cells and B cells that recognize the constant region and multimerization domains of the anti-idiotype (middle). A boost with an Env-derived immunogen specifically expands the bNAb precursor B cells that were preexpanded by the anti-idiotype-prime as well as some off-target Env specific cells (right). **(B)** The same initial pool of B cells (left) responds to immunization with Env by producing expanded clones of Env-reactive B cells including bNAb precursors and off-target B cells (middle). Boosting with another Env-derived immunogen amplifies both target and off-target B cells (right).

There are three potential advantages of this approach. First, any B cells expanded by anti-idiotype that express antibodies that do not react with Env will not be further expanded upon subsequent immunization with HIV-1 Env antigens (Fig. 7 A). In contrast, priming with a germline-targeting Env would, in addition to activating the target B cells, activate off-target B cell responses that would continue to compete upon Env boosting (Fig. 7 B). Second, the anti-idiotype approach does not require a priori structural understanding or specific engineering to produce a reagent that targets the inferred germline antibody-producing B cell. Third, this method could be applied to any vaccine for which there are shared antibody signatures that are associated with protection. For example, potent anti-Zika virus VH3-23/VK1-5 antibodies are found in many high-responder individuals, and bNAbs to influenza are typically VH1-69 (Sui et al., 2009; Corti et al., 2010; Ekiert et al., 2011; Robbiani et al., 2017).

In summary, the data indicate that anti-idiotypic antibodies that target specific germline BCRs can be used to activate and expand antigen-specific naive B cells that are present in a polyclonal repertoire. This strategy may complement HIV-1 Env–based immunization approaches and is potentially applicable to other vaccines where immunodominant off-target responses interfere with effective vaccine development.

## Materials and methods

### Hybridoma generation

We injected mice three times with germline VRC01 (VH1-2*02 IgH/VK3-11 IgL). 3 d after the final injection spleens were harvested and used to generate hybridomas at the Fred Hutchinson Antibody Technology Center. Hybridoma supernatants were initially screened against glVRC01 to identify antigen-specific hybridomas. Supernatants from positive wells were then screened against a small panel of monoclonal antibodies that included glVRC01, as well as other germline non-VRC01-class antibodies that served as isotype controls by ELISA. Wells containing hybridomas that displayed positive binding for glVRC01, but negative binding to isotype controls, were sub-cloned by limiting dilution and screened for binding to a larger panel of antibodies using Bio-Layer Interferometry (BLI) on the Octet Red96 (ForteBio). Anti-mouse IgG Fc capture sensors (ForteBio) were immersed in hybridoma supernatant for 200 s. After loading, the baseline signal was then recorded for 60 s in kinetics buffer (KB; 1× PBS, 0.01% BSA, 0.02% Tween 20, and 0.005% NaN_3,_ pH 7.4). The sensors were then immersed in KB containing 20 µg/ml of purified human antibody for a 500 s association step. The maximum response was determined by averaging the nm shift over the last 5 s of the association step after subtracting the background signal from each analyte-containing well using empty anti-mouse IgG Fc capture ensors sensors at each time point. The binding screen included germline and mature VRC01-class mAbs, non-VRC01-class mAbs, and chimeric mAbs comprised of either a VRC01-class IgH paired with a non-VRC01-class IgL, or a non-VRC01-class IgH paired with a VRC01-class IgL. Most hybridomas including iv1 bound glVRC01 or glNIH45-46 which differ only in the CDRH3 region, but showed no cross reactivity to other antibodies in the panel.

### Plasmids

The variable heavy (VH) and variable light (VL) sequences of the iv8 and iv1 hybridomas (GenBank accession nos. MK611925–MK611928) were recovered using the mouse iG primer set (Millipore) using the protocol outlined in Siegel (2009), and Sanger sequenced (Genewiz). The VH/VL sequences were codon-optimized and cloned into full-length pTT3 derived IgG1 and IgL kappa expression vectors containing human constant regions using Gibson assembly (Snijder et al., 2018). The iv8 and iv1 VH regions were also cloned in frame with the human IgG-C1 domain fused to the modified multimerization domain of the C4b binding protein (C4b) from *Gallus gallus* (Ogun et al., 2008) followed by a cathepsin cleavage site (Schneider et al., 2000) and the 2W1S peptide (Rees et al., 1999). Another variant of iv8-C4b was made without the C-terminal cathepsin-2W1S fusion. pTT3-426c-N276D-gp140-KO was generated by introducing the D368R and E370A mutations (McGuire et al., 2014a) into pTT3-426c-N276D gp140 (McGuire et al., 2013) plasmid using site directed mutagenesis according to the manufacturer’s recommendations (Agilent Quickchange XLII). pTT3-426c-N276D-C4b-2W1S was created by cloning cDNA corresponding to amino acids 1–479 of the 426c N276D from pTT3-426c-N276D gp140 in-frame with the C4b multimerization domain and 2W1S peptide described above. The coding sequence of eOD-GT8 (Jardine et al., 2015) preceded by a leader peptide (MDAMKRGLCCVLLLCGAVFVSPSAS) was synthesized by IDT DNA technologies and cloned into pTT3 followed by a gly-ser linker and a his-avi tag (McGuire et al., 2014a) to create pTT3 eOD-GT8-his-avi. pTT3 eOD-GT8-KO was generated by introducing the D276N, W277F, R278T, D279A, and D368R mutations into pTT3-eOD-GT8-his-avi by site directed mutagenesis.

### Antibody purification

Plasmids encoding mAb heavy and light chains were transfected into 293F cells at a density of 10^6^ cells ml^−1^ in Freestyle 293 media using the 293Free transfection reagent according to the manufacturer’s instructions or PEI. Expression was performed for 6 d, after which cells and cellular debris were removed by centrifugation at 4,000 ×*g* followed by filtration through a 0.22-µm filter. Clarified cell supernatant containing recombinant antibodies was passed over Protein A or G Agarose, followed by extensive washing with PBS, and then eluted with 1 ml of Pierce IgG Elution Buffer, pH 2.0, into 0.1 ml of Tris HCl, pH 8.0. Purified antibodies were then dialyzed overnight into PBS, and stored at −20°C before use.

### Binding screen with recombinant anti-idiotypic mAbs

To verify the specificity of recombinant iv8 and iv1, they were biotinylated at a theoretical 1:1 mAb to biotin ratio using the EZ-Link NHS-PEG4-Biotin kit (Thermo Fisher Scientific) and then subjected to the BLI screen described in the “hybridoma generation” section with the following change to the first capture step: iv8 or iv1 was immobilized on a streptavidin biosensor (ForteBio) by immersing the biosensor into a 10 µg/ml solution of biotinylated mAb.

### scFv expression and complex formation

scFvconstructs were cloned into the pTT3 plasmid vector. ScFvs contain only the Fv antibody domains of VH/VL connected by an amino acid linker GGGGSGGGGSGGGGS from the C-terminus of the light chain to the N-terminus of the heavy chain. Iv8 scFv and glVRC01 scFv were expressed using HEK293ENBA cells (Tom et al., 2008). Cells were cultured in suspension and transfected with 500 µg/liter plasmid DNA using 293 Free Transfection Reagent (Novagen). After 6 d, cells were centrifuged at 4,500 rpm for 20 min. Supernatant was filter sterilized and incubated with His60 Ni-Superflow Resin (Novagen) overnight at 4°C, capturing scFv’s with a 6his-tag on the C-terminus of the proteins. The Ni resin was separated from the supernatant and washed with a solution of 150 mM NaCl, 20 mM Tris (pH 8.0), 20 mM imidazole (pH 7.0) and eluted with a solution of 300 mM NaCl, 50 mM Tris pH 8.0, 250 mM imidazole (pH 7.0). To separate the scFv monomers from diabodies and triabodies and to further purify the proteins, the sample was run on size-exclusion chromatography using a HiLoad 16/600 Superdex 200 pg (GE) column. Complexes of iv8 scFv and glVRC01 scFv were formed by mixing proteins at a 1:1 molar ratio. ScFv complexes were removed from monomeric scFv using size-exclusion chromatography. Complexes were concentrated to ∼10 mg/ml for crystallization trials.

### Crystallization

Crystallization conditions for iv8scFv and glVRC01scFv complexes were screened and monitored with a Formulatrix NT8 drop setting and Rock Imager. Screening was done with Hampton Research Crystal Screen HT, Molecular Dimensions Proplex screen HT-96, and Rigaku Wizard Precipitant Synergy block no. 2 using the vapor diffusion method. Final crystals were grown in a solution of 0.13 M NaCl, 0.13 M Tris (pH 8.0), 10.4% PEG 20,000. Crystals were cryoprotected in solutions containing 30% molar excess of their original reagents and 20% ethylene glycol. Crystals diffracted to 2.42 Å. Data were collected at Advanced Light Source 5.0.2 and processed using HKL2000 (Otwinowski and Minor, 1997).

### Structure solution and refinement

The structure of iv8scFv-glVRC01scFv was solved by molecular replacement with Phaser in the CCP4 suite (Collaborative Computational Project, Number 4, 1994) using the glVRC01 Fv portion of PDB ID: 6MFT (Borst et al., 2018) as the search model for both iv8 and glVRC01. Model building and refinement were performed using COOT (Emsley and Cowtan, 2004) and Phenix (Adams et al., 2010). Statistics are summarized in Table S1.

### Measurement of iv8 binding to 3BNC60^SI^

Kinetic measurements were obtained on the Octet Red instrument at 30°C with shaking at 1,000 rpm. Recombinant humanized iv8 was immobilized on Anti-Human IgG Fc capture sensors (ForteBio). After loading, the baseline signal was then recorded for 60 s in KB. The sensors were then immersed into wells containing serial dilutions of purified recombinant 3BNC60^SI^ Fab for a 300 s association step. Sensors were then immersed in KB for 600 s to determine the dissociation step. The background signal from each analyte-containing well was measured using reference sensors loaded with a negative control antibody, and subtracted from the signal obtained with each corresponding ligand-coupled sensor at each time-point. Curve fitting was performed using a 1:1 binding model and the ForteBio data analysis software. Mean association rate (k_on_) and dissociation rate (k_off_) values were determined by averaging all binding curves that matched the theoretical fit with an R^2^ ≥0.98.

### Multimeric iv8 Fab purification

Plasmids encoding iv8-IgH (Fab)-C4b or iv8 IgH (Fab)-C4b-2W1S were cotransfected with plasmids of iv8-Igk (1:1) into Expi-293 cells (Invitrogen) according to the manufacturers’ instructions. The supernatants were collected after 5 d of culture, filtered through 0.45 µm filter units (Millipore), and diluted 1:1 in IgG binding buffer (Thermo Fisher Scientific). Multimeric iv8 Fab complexes were purified by Protein G Agarose beads (Thermo Fisher Scientific), concentrated in centrifugal filter (30-kD cutout; Amicon), buffer exchanged with TBS pH 5.5, and further purified using size exclusion chromatography (Superdex200, GE Healthcare). The multimeric state of iv8 Fab-C4b or iv8 Fab-C4b-2W1S were verified by nonreducing SDS-PAGE and the binding activity was verified by BLI.

### Recombinant protein expression

pTT3-426c-N276D-C4b-2W1S, pTT3-426c-N276D-gp140, pTT3-426c-N276D-gp140-KO, pTT3-eOD-GT8-his-avi, and pTT3-eOD-GT8-KO-his-avi expression plasmids were transfected into 293 F cells at a density of 10^6^ cells ml^−1^ in Freestyle 293 media (Life Technologies) using the 293Free transfection reagent (EMD Millipore). Expression was performed in Freestyle 293 media for 6 d with gentle shaking at 37°C in the presence of 5% CO_2_ after which cells and cellular debris were removed by centrifugation at 10,000 ×*g* followed by filtration through a 0.2 µM filter. Clarified cell supernatant containing 426c-N276D-C4b-2W1S, 426c-N276D-gp140, and pTT3-426c-N276D-gp140-KO was passed over Agarose-bound Galanthus Nivalis Lectin (GNL) resin (Vector Laboratories), preequilibrated with 20 mM Tris, 100 mM NaCl, 1 mM EDTA (pH 7.4; GNL binding buffer), followed by extensive washing with GNL binding buffer. Bound protein was eluted with GNL binding buffer containing 1 M methyl mannopyranoside. Clarified cell supernatant containing eOD-GT8-his-avi and eOD-GT8-his-avi, was passed over nickel nitrilotriacetic acid (Ni-NTA) resin preequilibrated with Ni-NTA binding buffer (0.3 M NaCl, 20 mM Tris, 10 mM imidazole [pH 8.0]), followed by extensive washing with Ni-NTA binding buffer, and then eluted with 250 mM imidazole, 0.3 M NaCl, 20 mM Tris (pH 8.0; Ni-NTA elution buffer). All affinity purified proteins were further purified using a 16/60 S200 size-exclusion column (GE Healthcare) preequilibrated in PBS. Fractions containing purified proteins were pooled, aliquoted, frozen in liquid nitrogen, and stored at −20°C.

### Mice

3BNC60 HCmt and HCgl knock-in mice (CD45.2^+^) carry the prerearranged *Ig* V(D)J genes encoding the mature (somatically mutated) or the predicted germline IgH respectively, of human bNAb 3BNC60. 3BNC60^SI^ knock-in mice (CD45.2^+^) carry the prerearranged *Ig* V(D)J genes encoding the mature IgH and predicted germline IgL of human bNAb 3BNC60 (Dosenovic et al., 2015; McGuire et al., 2016). Mice used in experiments were homozygous for knock-in alleles. B6.SJL (CD45.1^+^) mice were obtained from the Jackson Laboratory. All experiments were conducted at The Rockefeller University according to approved Institutional Animal Care and Use Committee protocols.

### Animal experiments

40 µg 2W1S peptide was immunized i.p. in CFA adjuvant (Sigma) according to manufacturers’ instructions 2–4 wk before adoptive transfer or antigen injection. CD43-depleted 3BNC60^SI^ B cells (MACS Miltenyi Biotec) were transferred intravenously into B6.SJL recipient mice. 10–30 µg of indicated protein (mAb iv8, iv8 Fab-2W1S, and Env N276D-2W1S nanoparticles or control antibodies) were injected i.p. or s.c. in Sigma Adjuvant System (Sigma-Aldrich; Ribi) according to manufacturers’ instructions.

### Murine lymphocyte preparation and adoptive transfer

Lymphocytes were harvested by forcing lymph nodes and/or spleen through 70 µm filters (BD) into RPMI 1640 media (Gibco) containing 6% fetal bovine serum (FBS) and 10 mM HEPES, followed by lysis of erythrocytes using 1X ACK Lysis Buffer (Gibco). B cells were enriched by negative selection using anti-CD43 MicroBeads (Militenyi Biotec) with magnetized LS columns according to manufacturers’ instructions. In some experiments, enriched B cells were labeled with CellTrace Violet (CTV; Thermo Fisher Scientific) according to manufacturers’ instructions. Upon analysis of 3BNC60^SI^ plasmablast- and early memory B cell differentiation, >2 × 10^6^ CTV-labeled 3BNC60^SI^ B cells were transferred i.v. Approximately 100,000 B cells are then present in the mouse according to calculations in (Dosenovic et al., 2018) where ∼5% of the transferred B cells were shown to survive. These high numbers of B cells are needed in order to be accurately detect at early times after injection. In experiments where adoptively transferred 3BNC60^SI^ cells or serum responses were analyzed at later time points (>10 d), 500,000 enriched naive 3BNC60^SI^ B cells were transferred i.v., resulting in 25,000 naive 3BNC60^SI^ B cells/mouse. When total 3BNC60^SI^ GC cells and plasmablasts per lymph node were determined, the average number of cells found in two lymph nodes collected from the same animal was used in the plots.

### Flow cytometry and single-cell sorting of murine cells

For murine B cell sorting, single cell suspensions of lymphocytes were maintained at 4°C in FACS buffer (PBS containing 2% FBS and 1 mM EDTA). Fc receptors were blocked using rat anti-mouse CD16/32 (clone 2.4G2; BD). Cells were stained with fluorophore-conjugated antibodies to: B220, IgM, CD38, CD45.1, CD45.2 (eBioscience), IgD, CD138 (BioLegend) GL7, CD95, CD4, CD8, NK1.1, Gr1, or F4/80 (BD). Dump staining including CD4, CD8, NK1.1, Gr1, and F4/80 were included in all flow cytometry stainings. Dead cells were excluded from analyses using Zombie NIR Fixable Viability Kit (BioLegend). Intracellular Env-specific B cells were stained using biotinylated Avi-tagged monomeric (426c.TM4ΔV1-3 [McGuire et al., 2016]; 5 µg/ml) and detected using Streptavidin-PE (BD) following manufacturers instructions for intracellular staining. iv8-binding cells were identified by staining the cells with biotinylated iv8 antibody and detected using Streptavidin-PE (BD). Total number of cells were calculated using AccuCheck Counting Beads according to the manufacturers’ description (Thermo Fisher Scientific). Samples were acquired on a BD Fortessa or BD Symphony and analyzed using FlowJo software (TreeStar). In single-cell sorting experiments, naive Env-binding cells of 3BNC60 HCmt mice were individually sorted based on CD4^−^, CD8^−^, Gr-1^−^, F4/80^−^, NK1.1^−^, B220^+^, IgM^+^, 426c.TM4ΔV1-3^+^ expression. Memory B cells from iv8-injected 3BNC60 HCmt mice were single cell–sorted based on CD4^−^, CD8^−^, Gr-1^−^, F4/80-, NK1.1^−^ B220^+^, CD38^+^, IgM^−^, IgG1^+^, 426c.TM4ΔV1-3^+^ expression. IgG1^+^ GC B cells were single cells sorted based on CD4^−^, CD8^−^, Gr-1^−^, F4/80^−^, NK1.1^−^, B220^+^, CD95^+^, CD38^−^, IgG1^+^ expression. Cells were single cell–sorted into 96-well plates using a FACSAria III sorter (Becton Dickinson). The cells were lysed with 4 µl lysis buffer containing RNasin (Promega) in 40 U/µl (0.3 µl), 10× Dulbecco's PBS (DPBS; 0.2 µl), dithiothreitol (Invitrogen) 100 mM (0.4 µl), and nuclease-free water (3.1 µl). The sorted plates were stored at −80°C until further processing (Tiller et al., 2009). IgL sequences were retrieved using primers described in (Dosenovic et al., 2015). A clone was defined as nucleotide sequences with the same IgVL and IgJL germline gene and similar CDRL3 nucleotide sequences (similarity of CDRL3 was defined using hamming distance threshold of 0.15). Samples were acquired on a BD FACSAria.

### ELISA

Corning 3690 half-well 96-well plates were coated overnight at 4°C with 50 µl/well of 2 µg/ml eOD-GT8, eOD-GT8-KO, 426cTM_4_ΔV1-3, 426cTM_4_ΔV1-3-KO, 426c Env N276D trimer, 426c Env N276D-KO trimer or murine iv8 Fab in PBS. Plates were washed six times in PBS 0.05% Tween 20 (wash buffer). Plates were blocked with 100 µl/well PBS 5% milk (blocking buffer) for 2 h at room temperature (RT). Sera or monoclonal antibodies were prepared at 1:30 dilution or a concentration of 10 µg/ml in fresh blocking buffer, respectively, and further diluted in threefold or fivefold serial dilutions, respectively. A total volume of 50 µl was added to the plates and incubated for 2 h at RT. Binding was revealed by either anti-mouse IgG-HRP (Jackson ImmunoResearch) or anti-human IgG-HRP (Jackson ImmunoResearch) diluted 1:5,000 in wash buffer. Plates were incubated for 1 h at RT. Plates were washed six times in wash buffer. HRP activity was determined using ABTS (2,2′-azinobis [3-ethylbenzothiazoline-6-sulfonic acid]-diammonium salt) substrate solution (Life Technologies), adding 50 µl/well. Plates were read at 405 nm on a FLUOstar Omega microplate reader (BMG Labtech). Data were analyzed with Microsoft Excel and GraphPad Prism 6.0.

### Preparation of zenon mAb labeling decoys

Zenon APC-DL755 was generated by conjugating the Zenon APC Human IgG labeling reagent, from the Zenon Allophycocyanin Human IgG Labeling Kit (Z25451) to DyLight 755 NHS Ester (62279; Thermo Fisher Scientific) according to manufacturers’ instructions. Zenon PE-DL650, was generated by conjugating the Zenon PE Human IgG labeling reagent from the Zenon R-Phycoerythrin Human IgG Labeling Kit (Z25455) with DyLight 650 NHS Ester (62266; Thermo Fisher Scientific) according to the manufacturers’ instructions. To create decoy reagents, Zenon APC-DL755 and Zenon PE-DL650 were incubated with Zenon blocking reagent at a 1:1 ratio.

### Fluorescent iv8 probes

To generate iv8-APC, 1 µg of iv8 was incubated with 5 µl Zenon APC Human IgG labeling reagent, and incubated at RT for 10 min, then incubated with 5 µl of Zeonon blocking reagent and stored at RT until use. To generate iv8-PE, 1 µg of iv8 was incubated with 5 µl Zenon PE Human IgG labeling reagent, and incubated at RT for 10 min, then incubated with 5 µl of Zenon blocking reagent and stored RT until use.

### Human subjects

Peripheral blood mononuclear cells (PBMCs) and serum were collected from HIV-uninfected adults recruited at the Seattle HIV Vaccine Trials Unit (Seattle, Washington) as part of the study “Establishing Immunologic Assays for Determining HIV-1 Prevention and Control,” also referred to as Seattle Assay Control. All participants signed informed consent, and the following institutional human subjects review committee approved the protocol before study initiation: Fred Hutchinson Cancer Research Center institutional review board (Seattle, Washington). PBMC samples from donors were blindly selected at random with no considerations made for age or sex.

### Human B cell sorting

Cryopreserved PBMC were thawed and resuspended in 200 µl of easysep buffer (1× PBS, 2% heat inactivated fetal bovine serum, 1 µM EDTA). B cells were isolated using the StemCell Human B Cell Enrichment Kit (19054; StemCell Technologies) according to the manufacturers’ instructions. Enriched B cells were resuspended in 200 µl EasySep buffer and incubated with 10 µl rat serum, 10 µl mouse serum, 10 µl mouse–anti-human CD32 (551900; BD), 10 µl mouse–anti-human CD23 (555707; BD), and 10 µl mouse–anti-human CD16 (550383; BD). Cells were then washed with EasySep buffer and resuspended in 200 µl EasySep buffer and incubated with 5 µl of Zenon APC-DL755 and 5 µl of Zenon PE-DL650 and incubated on ice for 10 min. Next, B cells were stained with iv8 conjugated to either Zenon-PE or Zenon-APC and CD19-BV711 (302246; BioLegend) at 1:200 dilution, CD27-PECy7 (25-0271-82; eBioscience ) at a 1:600 dilution, CD14- FITC (557153; BD) at 1:60 dilution, CD3-FITC (556611; BD) at a 1:60 dilution, CD20-AF700 (302322; BioLegend) at a 1:300 dilution, IgD-PerCP-Cy5.5 (46-9868-42; eBioscience) at a 1:120 dilution, IgM-BV605 (314524; BioLegend) at a 1:120 dilution, and Fixable Viability Dye eFluor 506 (65-0866-14; eBioscience) at a 1:300 dilution. Cells were then washed with 5 ml of EasySep buffer and suspended in 0.5 ml of EasySep buffer. Cells were analyzed by flow cytometry using a BD FACS Aria II. Naive iv8^+^ B cells were defined as live (eFluor 506^−^), CD14^−^, CD3^−^, CD19^+^, CD20^+^, IgM^+^, IgD^+^, CD27^−^, APC-DL755^−^, PE-DL650^−^, PE^+^, APC^+^. Naive iv8^+^ B cells were single-cell sorted into 96-well plates. cDNA was generated using iScript (Bio-Rad) and the VH and VK sequences were recovered using gene specific primers and cycling conditions previously described (Tiller et al., 2009). Alternatively, naive iv8^+^ B cells were bulk-sorted and sequenced using the human B Cell V(D)J Enrichment Kit on the Chromium platform (10X Genomics). As a control, naive unsorted B cells were isolated using the EasySep Human B cell isolation kit (Stemcell Technologies). BCR sequences were obtained using the using the human B Cell V(D)J Enrichment Kit on the Chromium platform (10X Genomics). Because iv8 strongly selects for kappa light chains, lambda light chain transcripts were excluded from analysis of the 10X sequencing data.

### Statistical analysis

Data were analyzed with Prism 6 (GraphPad) for normal distribution using the D’Agostino-Pearson normality test. Data points that displayed normal Gaussian distribution were further analyzed for significant differences with Prism 6 (GraphPad) using two-tailed unpaired Student’s *t* tests. Data points that did not display a normal Gaussian distribution were analyzed for statistical differences using the Mann-Whitney *U* test. Data were considered significant at *, P ≤ 0.05; **, P ≤ 0.01, ***, P ≤ 0.001.

### Online supplemental material

Fig. S1 depicts flow cytometric gating strategies for enumeration of B cells after injection of iv8 Fab-2W1S and N276D-2W1S nanoparticles. Fig. S2 depicts flow cytometry gating strategy for single cell sorting of heavy chain knock-in mice. Table S1 describes the data collection and refinement statistics for iv8scFv bound to glVRC01scFv. Table S2 describes the molecular interactions between glVRC01 and iv8 in detail. Table S3 contains the kinetic parameters of iv8-binding to 3BNC60^SI^ determined by BLI. Table S4 contains BCR sequence data obtained from control-injected 3BNC60 HCgl knock-in mice. Table S5 contains BCR sequence data obtained from iv8 Fab-2W1S nanoparticle-injected 3BNC60 HCgl knock-in mice. Table S6 contains BCR sequence data information from HIV-1 Env-binding naive B cells in a 3BNC60 HCmt knock-in mouse. Table S7 contains BCR sequence data from HIV-1 Env binding B cells in a 3BNC60 HCmt knock-in mouse after injection with iv8.

## Supplementary Material

Supplemental Materials (PDF)

Table S4 (Excel file)

Table S5 (Excel file)

Table S6 (Excel file)

Table S7 (Excel file)
