# Supplementary material for: Anti-idiotypic antibodies elicit anti-HIV-1–specific B cell responses
Source: J Exp Med. 2019 Jul 25;216(10):2316–30. doi: 10.1084/jem.20190446 (PMC6780999; doi:10.1084/jem.20190446)
Supplement: Supplemental Materials (PDF) [file JEM_20190446_sm.pdf]

Supplemental material

Dosenovic et al., https://doi.org/10.1084/jem.20190446

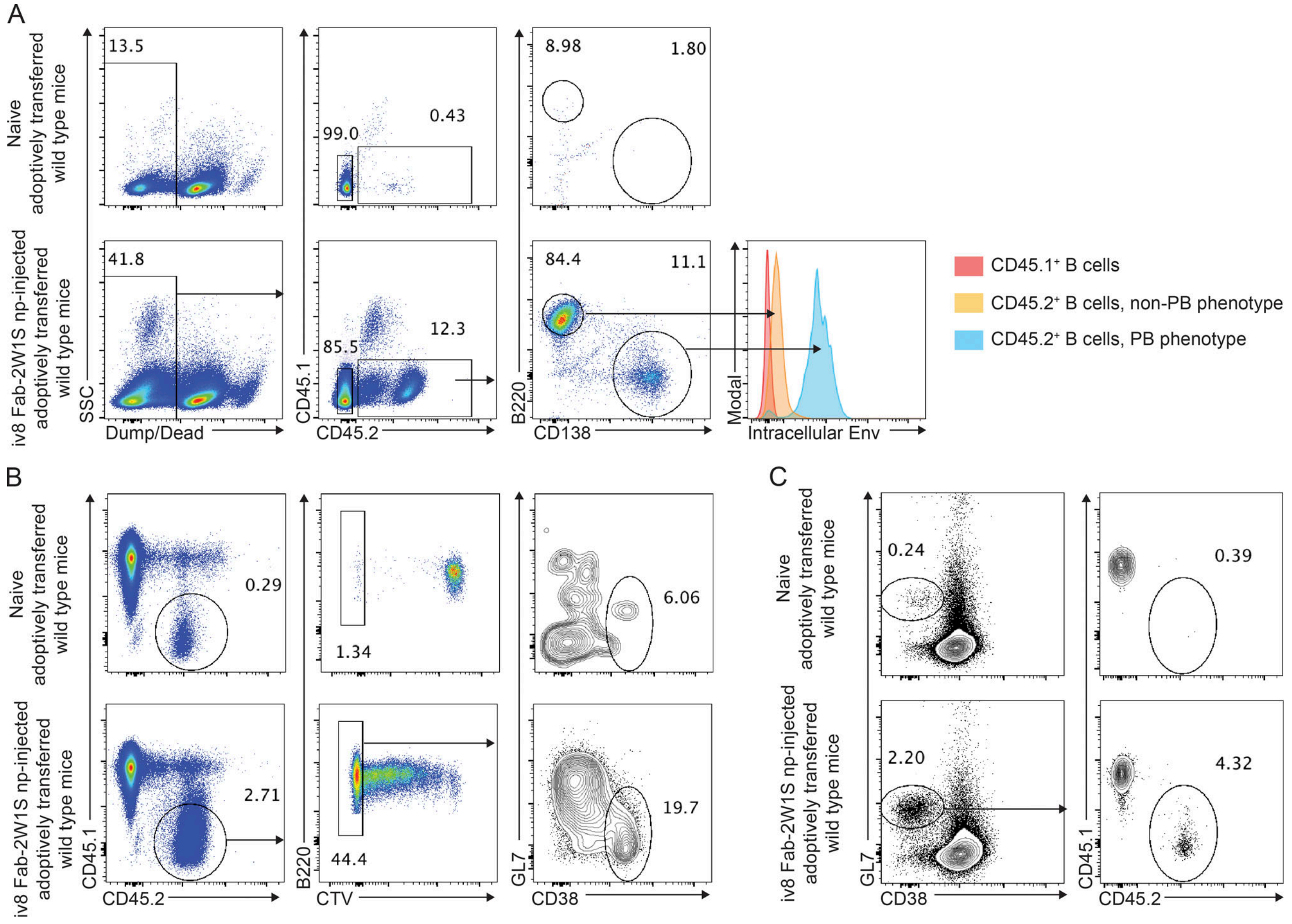

Figure S1. **Flow cytometric gating strategies for enumeration of B cells after injection of iv8 Fab-2W1S and N276D-2W1S nanoparticles. (A)** Gating strategy for enumeration of 3BNC60^{SI} plasmablasts (PB; CD45.2$^+$, CD138$^+$, B220$^-$, intracellular Env^{high}) in adoptively transferred wild-type mice 5 d after injection of iv8 Fab-2W1S and N276D-2W1S nanoparticles. Cells were pregated on live lymphocytes and singlets. SSC, side scatter. **(B)** Gating strategy for enumeration of 3BNC60^{SI} early memory B cells (mB; CD45.2$^+$, CTV$^-$, CD38$^+$, GL7$^-$) in adoptively transferred wild-type mice 5 d after injection of iv8 Fab-2W1S and N276D-2W1S nanoparticles. Cells were pregated on lymphocytes, singlets, and live B220$^+$ B cells. **(C)** Gating strategy for enumeration of 3BNC60^{SI} GC cells (CD45.2$^+$, CD95$^+$, CD38$^-$) in adoptively transferred wild-type mice. Cells were pregated on lymphocytes, singlets, and live B220$^+$ B cells.

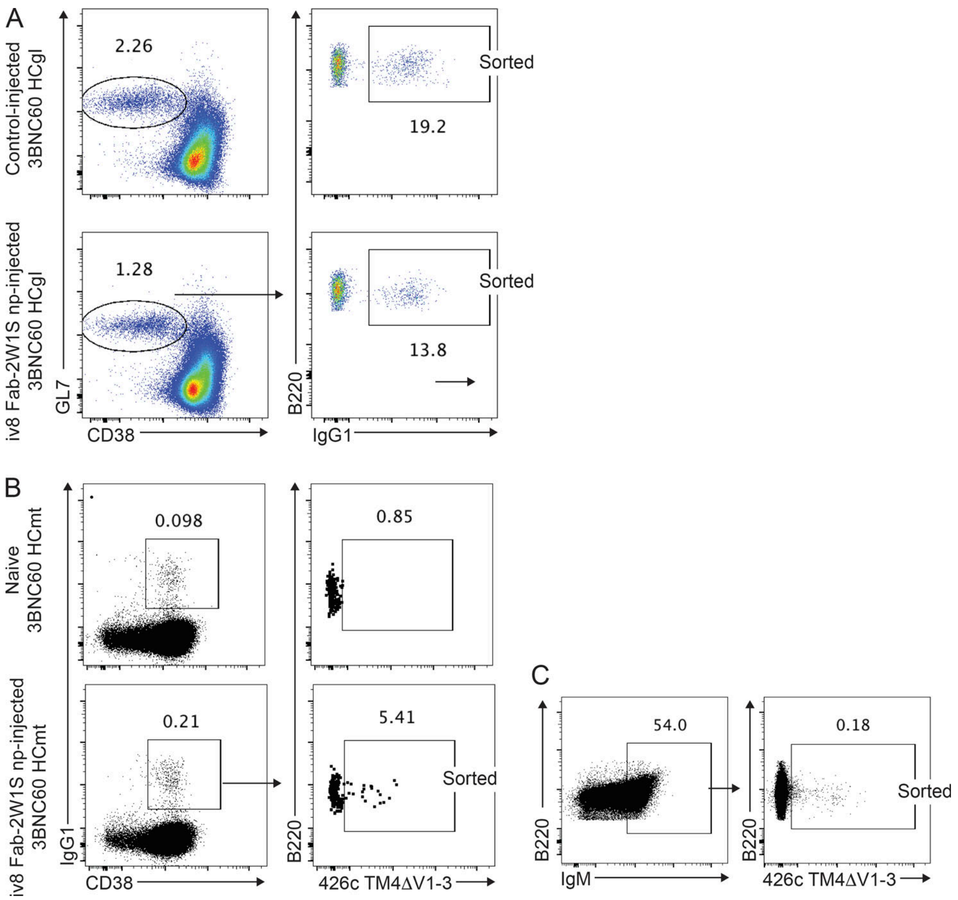

Figure S2. **Flow cytometry gating strategy for single-cell sorting of heavy-chain knock-in mice. (A)** Flow cytometry gating strategy for single-cell sorting of IgG1⁺ GC B cells 14 d after injection of 3BNC60 HCgl mice with control iv1 Fab-2W1S nanoparticles (top panels) or iv8 Fab-2W1S nanoparticles (bottom panels). Cells were pregated on lymphocytes, singlets, or live B220⁺ B cells. **(B)** Flow cytometry gating strategy for the identification of Env-specific (426c TM4ΔV1–3) memory B cells in naive (top panels) or iv8 Fab-2W1S nanoparticle-injected 3BNC60 HCmt mice (bottom panels) 42 d after injection. Cells were pregated on lymphocytes, singlets, live B cells, or IgM⁻. **(C)** Flow cytometry gating for the isolation of single naive B cells from 3BNC60 HCmt mice binding to HIV-1 Env (426c TM4ΔV1–3). Cells were pregated on lymphocytes, singlets, or live B cells.

Table S1. **Data collection and refinement statistics for iv8scFv + glVRC01scFv**

**Data collection**	
Space group	P1
Cell dimensions	
a, *b*, *c* (Å)	51.583, 107.937, 116.961
α, *β*, *γ* (°)	85.489, 80.276, 84.172
Resolution (Å)	50–2.419 (2.506–2.42)[a]
R_{sym} or R_{merge}	0.059 (0.169)[a]
I/s*I*	10.2 (2.8)[a]
Completeness (%)	92.0 (50.2)[a]
Redundancy	1.7 (1.4)[a]
*CC*1/2	(0.952)[a]
**Refinement**	
Resolution (Å)	49.78–2.419 (2.506–2.419)[a]
No. reflections	87,138 (5,909)
R_{work}/R_{free} (%)	17.43/22.40 (21.65/27.10)[a]
No. atoms	15,691
Protein	14,355
Water	1,152
Ligand	184
B-factors (Å^2)	33.82
Protein	33.35
Water	36.56
Ligand	53.41
Root mean square deviations	
Bond lengths (Å)	0.003
Bond angles (°)	0.96
Ramachadran favored %	96.98
Ramachadran outliers %	0.00
MolProbity all-atoms clashscore	3.25
**Protein Data Bank ID**	6OL7

$R_{sym} = R_{merge} = [\sum_h \sum_i |I_h - I_{hi}| / \sum_h \sum_i I_{hi}]$ where I_h is the mean of I_{hi} observations of reflections *h*. $R_{work} = \sum ||F_{obs}| - |F_{calc}|| / \sum |F_{obs}|$ for 95% of recorded data. $R_{free} = \sum ||F_{obs}| - |F_{calc}|| / \sum |F_{obs}|$ for 5% of recorded data. *I*/s*I*, signal to noise ratio.
[a]Statistics for the highest resolution shell are shown in parentheses.

Table S2. Detailed interactions between glVRC01 and iv8

a. Detailed interactions of glVRC01 light chain with iv8 heavy chain

<table>
<tr><th>glVRC01 Light Chain</th><th>HSDC</th><th>ASA</th><th>BSA</th></tr>
<tr><td>L:SER 28</td><td>H</td><td>89.59</td><td>23.35 |||</td></tr>
<tr><td>L:VAL 29</td><td></td><td>64.20</td><td>0.49 |</td></tr>
<tr><td>L:SER 30</td><td></td><td>96.21</td><td>31.64 ||||</td></tr>
<tr><td>L:SER 31</td><td></td><td>30.91</td><td>12.67 |||||</td></tr>
<tr><td>L:TYR 32</td><td>H</td><td>165.01</td><td>68.50 |||||</td></tr>
<tr><td>L:TYR 49</td><td></td><td>102.29</td><td>0.78 |</td></tr>
<tr><td>L:ASP 50</td><td>H</td><td>60.26</td><td>34.24 ||||||</td></tr>
<tr><td>L:ALA 51</td><td></td><td>18.98</td><td>11.80 |||||||</td></tr>
<tr><td>L:SER 52</td><td>H</td><td>66.81</td><td>51.92 ||||||||</td></tr>
<tr><td>L:ASN 53</td><td>H</td><td>55.62</td><td>43.51 ||||||||</td></tr>
<tr><td>L:GLY 64</td><td></td><td>8.20</td><td>0.61 |</td></tr>
<tr><td>L:SER 65</td><td></td><td>62.92</td><td>11.05 ||</td></tr>
<tr><td>L:GLY 66</td><td>H</td><td>43.27</td><td>34.64 |||||||||</td></tr>
<tr><td>L:SER 67</td><td></td><td>84.67</td><td>17.84 |||</td></tr>
<tr><td>L:GLY 68</td><td>H</td><td>65.72</td><td>57.00 |||||||||</td></tr>
<tr><td>L:THR 69</td><td></td><td>40.69</td><td>0.84 |</td></tr>
<tr><td>L:PHE 71</td><td></td><td>20.20</td><td>10.01 |||||</td></tr>
<tr><td>L:TYR 91</td><td></td><td>130.01</td><td>19.46 ||</td></tr>
</table>

<table>
<tr><th>iv8 Heavy Chain</th><th>HSDC</th><th>ASA</th><th>BSA</th></tr>
<tr><td>A:ALA 33</td><td></td><td>24.26</td><td>2.51 ||</td></tr>
<tr><td>A:ASN 52</td><td>H</td><td>43.98</td><td>36.57 |||||||||</td></tr>
<tr><td>A:ASN 52A</td><td>H</td><td>56.14</td><td>32.65 ||||||</td></tr>
<tr><td>A:ASN 53</td><td>H</td><td>120.69</td><td>37.55 ||||</td></tr>
<tr><td>A:GLY 55</td><td>H</td><td>72.55</td><td>54.07 ||||||||</td></tr>
<tr><td>A:TYR 56</td><td>H</td><td>120.29</td><td>71.62 ||||||</td></tr>
<tr><td>A:THR 57</td><td>H</td><td>53.73</td><td>28.53 ||||||</td></tr>
<tr><td>A:HIS 58</td><td></td><td>90.31</td><td>47.65 ||||||</td></tr>
<tr><td>A:TYR 59</td><td></td><td>23.23</td><td>1.61 |</td></tr>
<tr><td>A:TYR 97</td><td>H</td><td>154.18</td><td>24.79 ||</td></tr>
<tr><td>A:TRP 98</td><td></td><td>213.44</td><td>100.02 |||||</td></tr>
</table>

Hydrogen Bonds

glVRC01 Light Chain	Dist. [Å]	iv8 Heavy Chain
L:ASP 50[O]	3.17	A:ASN 52[ND2]
L:SER 52[OG]	2.88	A:ASN 52[ND2]
L:ASN 53[OD1]	2.86	A:ASN 52A[ND2]
L:SER 52[O]	2.69	A:ASN 53[ND2]
L:GLY 68[O]	2.87	A:TYR 56[OH]
L:GLY 66[O]	3.39	A:THR 57[N]
L:SER 28[OG]	2.99	A:HIS 58[NE2]
L:ASN 53[ND2]	3.13	A:ASN 52A[OD1]
L:GLY 66[N]	2.79	A:GLY 55[O]
L:GLY 68[N]	2.99	A:THR 57[O]
L:TYR 32[OH]	3.77	A:TYR 97[O]

b. Detailed interactions of glVRC01 light chain with iv8 light chain

<table>
<tr><th>glVRC01 Light Chain</th><th>HSDC</th><th>ASA</th><th>BSA</th></tr>
<tr><td>L:ILE 2</td><td></td><td>10.48</td><td>4.53 |||||</td></tr>
<tr><td>L:GLN 27</td><td></td><td>101.49</td><td>28.48 |||</td></tr>
<tr><td>L:SER 28</td><td>H</td><td>89.59</td><td>56.79 |||||||</td></tr>
<tr><td>L:VAL 29</td><td></td><td>64.20</td><td>57.48 |||||||||</td></tr>
<tr><td>L:SER 30</td><td>H</td><td>96.21</td><td>64.57 |||||||</td></tr>
<tr><td>L:SER 31</td><td></td><td>30.91</td><td>0.58 |</td></tr>
<tr><td>L:TYR 91</td><td>H</td><td>130.01</td><td>58.16 |||||</td></tr>
<tr><td>L:GLU 96</td><td>H</td><td>135.97</td><td>33.54 |||</td></tr>
<tr><td>L:PHE 97</td><td></td><td>44.92</td><td>12.23 |||</td></tr>
</table>

<table>
<tr><th>iv 8 Light Chain</th><th>HSDC</th><th>ASA</th><th>BSA</th></tr>
<tr><td>Q:GLN 27</td><td></td><td>87.15</td><td>5.15 |</td></tr>
<tr><td>Q:TYR 27D</td><td>H</td><td>94.17</td><td>77.59 |||||||||</td></tr>
<tr><td>Q:SER 27E</td><td></td><td>97.78</td><td>3.27 |</td></tr>
<tr><td>Q:GLY 27F</td><td></td><td>58.07</td><td>6.89 ||</td></tr>
<tr><td>Q:ASN 28</td><td></td><td>60.44</td><td>8.80 ||</td></tr>
<tr><td>Q:TYR 32</td><td>H</td><td>34.96</td><td>19.92 ||||||</td></tr>
<tr><td>Q:PHE 91</td><td>H</td><td>45.37</td><td>7.31 ||</td></tr>
<tr><td>Q:TYR 92</td><td>H</td><td>76.06</td><td>58.04 ||||||||</td></tr>
<tr><td>Q:PHE 93</td><td></td><td>60.83</td><td>37.01 |||||||</td></tr>
<tr><td>Q:TYR 94</td><td>H</td><td>169.30</td><td>68.44 |||||</td></tr>
<tr><td>Q:TRP 96</td><td>H</td><td>122.56</td><td>7.44 |</td></tr>
</table>

Hydrogen Bonds

glVRC01 Light Chain	Dist. [Å]	iv 8 Light Chain
L:SER 30[N]	2.85	Q:TYR 92[O]
L:SER 30[OG]	2.76	Q:PHE 91[O]
L:TYR 91[OH]	2.65	Q:TYR 32[OH]
L:GLU 96[N]	3.32	Q:TYR 27D[OH]
L:SER 28[O]	3.13	Q:TYR 94[N]
L:SER 30[OG]	2.73	Q:TRP 96[NE1]
L:GLU 96[OE2]	2.72	Q:TYR 27D[OH]

c. Detailed interactions of glVRC01 heavy chain with iv8 light chain

<table>
<tr><th>glVRC01 Heavy Chain</th><th>HSDC</th><th>ASA</th><th>BSA</th></tr>
<tr><td>H:TRP 47</td><td></td><td>64.81</td><td>4.53 |</td></tr>
<tr><td>H:TRP 50</td><td>H</td><td>51.04</td><td>30.75 |||||||</td></tr>
<tr><td>H:THR 57</td><td></td><td>55.30</td><td>7.13 ||</td></tr>
<tr><td>H:ASN 58</td><td>H</td><td>67.19</td><td>47.27 ||||||||</td></tr>
<tr><td>H:TRP 100B</td><td></td><td>114.31</td><td>47.15 |||||</td></tr>
</table>

<table>
<tr><th>iv8 Light Chain</th><th>HSDC</th><th>ASA</th><th>BSA</th></tr>
<tr><td>Q:TYR 27D</td><td></td><td>94.17</td><td>16.58 ||</td></tr>
<tr><td>Q:SER 27E</td><td>H</td><td>97.78</td><td>11.85 ||</td></tr>
<tr><td>Q:GLY 27F</td><td>H</td><td>58.07</td><td>51.18 |||||||||</td></tr>
<tr><td>Q:ASN 28</td><td></td><td>60.44</td><td>23.07 ||||</td></tr>
<tr><td>Q:GLN 29</td><td></td><td>147.98</td><td>46.23 ||||</td></tr>
</table>

Hydrogen Bonds

glVRC01 Heavy Chain	Dist. [Å]	iv8 Light Chain
H:ASN 58[ND2]	2.70	Q:SER 27E[O]
H:ASN 58[ND2]	3.02	Q:GLY 27F[O]
H:TRP 50[NE1]	2.75	Q:GLY 27F[O]

d. Detailed interactions of glVRC01 heavy chain with iv8 heavy chain

<table>
<tr><th>glVRC01 Heavy Chain</th><th>HSDC</th><th>ASA</th><th>BSA</th></tr>
<tr><td>H:ASP 99</td><td>H</td><td>130.70</td><td>31.05 |||</td></tr>
<tr><td>H:TYR 100</td><td></td><td>123.36</td><td>33.83 |||</td></tr>
</table>

<table>
<tr><th>iv8 Heavy Chain</th><th>HSDC</th><th>ASA</th><th>BSA</th></tr>
<tr><td>A:TYR 97</td><td>H</td><td>154.18</td><td>52.44 ||||</td></tr>
<tr><td>A:TRP 98</td><td></td><td>213.44</td><td>20.27 |</td></tr>
</table>

Hydrogen Bonds

glVRC01 Heavy Chain	Dist. [Å]	iv8 Heavy Chain
H:ASP 99[O]	2.49	A:TYR 97[OH]

HSDC, hydrogen/disulphide bond, salt bridge or covalent link**;** ASA, accessible surface area, Å²; BSA, buried surface area, Å²;
||||, buried area percentage, one bar per 10%.

Table S3. **Kinetics of iv8-binding to 3BNC60^{SI} determined by BLI**

K_D (M)	k_{on} (M^{-1} s^{-1})	k_{on} error	k_{off} (s^{-1})	k_{off} error
2.11×10^{-9}	1.26×10^{5}	9.10×10^{2}	2.66×10^{-4}	2.02×10^{-6}

K_D, dissociation constant; k_{on}, association rate; k_{off}, dissociation rate.

Tables S4–S7 are provided online as separate Excel files. Table S4 list IgL sequences from two single cell–sorted control-injected 3BNC60 HCgl knock-in mice. Table S5 lists IgL sequences from two single cell–sorted iv8 Fab-2W1S–injected 3BNC60 HCgl mice. Table S6 lists IgL sequences from one single cell–sorted naive 3BNC60 HCmt mouse. Table S7 lists IgL sequences from one single cell–sorted iv8 Fab-2W1S–injected 3BNC60 HCmt mouse.
